# A qualitative assessment of the context and enabling environment for the control of *Taenia solium* infections in endemic settings

**DOI:** 10.1371/journal.pntd.0009470

**Published:** 2021-06-11

**Authors:** Nicholas Ngwili, Nancy Johnson, Raphael Wahome, Samuel Githigia, Kristina Roesel, Lian Thomas

**Affiliations:** 1 Animal and Human Health Program, International Livestock Research Institute, Nairobi, Kenya; 2 College of Agriculture and Veterinary Sciences, University of Nairobi, Nairobi, Kenya; 3 CGIAR Research program on Agriculture for Nutrition and Health, IFPRI, Washington, DC, United States of America; 4 Institute for Parasitology and Tropical Veterinary Medicine, Freie Universität Berlin, Berlin, Germany; 5 Institute of Infection, Veterinary and Ecological Sciences, University of Liverpool, Lea Hurst Campus, Neston, United Kingdom; Federal University of Ceará, Fortaleza, Brazil, BRAZIL

## Abstract

**Background:**

*Taenia solium* (*T*. *solium*), is a zoonotic helminth causing three diseases namely; taeniasis (in humans), neurocysticercosis (NCC, in humans) and porcine cysticercosis (PCC, in pigs) and is one of the major foodborne diseases by burden. The success or failure of control options against this parasite in terms of reduced prevalence or incidence of the diseases may be attributed to the contextual factors which underpin the design, implementation, and evaluation of control programmes.

**Methodology/Principal findings:**

The study used a mixed method approach combining systematic literature review (SLR) and key informant interviews (KII). The SLR focused on studies which implemented *T*. *solium* control programmes and was used to identify the contextual factors and enabling environment relevant to successful inception, planning and implementation of the interventions. The SLR used a protocol pre-registered at the International prospective register of systematic reviews (PROSPERO) number CRD42019138107 and followed PRISMA guidelines on reporting of SLR. To further highlight the importance and interlinkage of these contextual factors, KII were conducted with researchers/implementers of the studies included in the SLR. The SLR identified 41 publications that had considerations of the contextual factors. They were grouped into efficacy (10), effectiveness (28) and scale up or implementation (3) research studies. The identified contextual factors included epidemiological, socioeconomic, cultural, geographical and environmental, service and organizational, historical and financial factors. The enabling environment was mainly defined by policy and strategies supporting *T*. *solium* control.

**Conclusion/Significance:**

Failure to consider the contextual factors operating in target study sites was shown to later present challenges in project implementation and evaluation that negatively affected expected outcomes. This study highlights the importance of fully considering the various domains of the context and integrating these explicitly into the plan for implementation and evaluation of control programmes. Explicit reporting of these aspects in the resultant publication is also important to guide future work. The contextual factors highlighted in this study may be useful to guide future research and scale up of disease control programmes and demonstrates the importance of close multi-sectoral collaboration in a One Health approach.

## Author summary

*Taenia solium*, commonly known as the pork tapeworm is a parasite that, as the etiological agent of neurocysticercosis (the presence of the intermediate state/cysts of the parasite in the central nervous system of humans) is one of the leading causes of acquired epilepsy across endemic areas of sub-Saharan Africa, Southern America and south-east Asia. A variety of biologically efficacious control options exist which include; deworming, provision of clean water and sanitation and appropriate hygiene and food safety education in humans and the vaccination, deworming, confinement and proper feeding of pigs. Despite these, the parasite has not been eliminated and varying impact of interventions has been achieved in the many endemic areas. Failure of the interventions to achieve expected impact could be attributed either to inadequate consideration of various contextual factors, or inability to create an enabling environment needed to underpin success. Contextual factors in this instance are features of the circumstances for which an intervention is conceived and developed and in which it is implemented and evaluated. We conducted a systematic literature review coupled with key informant interviews to identify these contextual factors and show how they may have influenced the implementation and evaluation of control programmes. The study identified that choice of baseline and end line measures, local knowledge, attitude and perceptions of the target population, existing infrastructural and staff capacities, stakeholder participation and support by the national governments through policies and strategies may affect the implementation and evaluation of control interventions. During project inception, there is need to document and understand these contextual factors in order to anticipate the challenges and adapt accordingly.

## Introduction

The zoonotic parasite *Taenia solium* (*T*. *solium*) and its associated diseases; taeniasis, porcine cysticercosis (PCC) and neurocysticercosis (NCC), are diseases of great public health and economic significance [1–3]. The parasite is among the neglected tropical diseases (NTDs)—diseases often associated with resource-constrained, marginalized and vulnerable populations, where poor animal husbandry may be practiced, and where there is limited access to clean water and sanitation [4, 5]. The 2012 ‘roadmap’, endorsed by the World Health Assembly in 2013, called for action to prevent, control, eliminate or eradicate NTDs including *T*. *solium* cysticercosis by 2020. The 2020 milestones of “Interventions scaled up in selected countries for *T*. *solium* taeniasis/cysticercosis control and elimination” has not been met. The updated 2021–2030 roadmap now provides specific targets for a number of countries with “intensified control in hyperendemic areas” [6].

Several control options focusing on human treatment, pig vaccination and treatment, health education, and/or sanitation exist for the control and eventual elimination of *T*. *solium* infections. Combinations of any of the listed options have been tested with varying degrees of success [7, 8]. As each component of the ‘toolkit’ of control options has either been demonstrated to be effective under experimental conditions or has a strong biological plausibility, we hypothesize that the success or failure of the interventions as measured by significant reduction in human or porcine prevalence/incidence of disease, may be attributed to the interaction between the intervention and factors present in the context within which they were planned, implemented and evaluated.

Context can be defined as the features of the circumstances for which an intervention is conceived and developed and in which it is implemented and evaluated [9]. Context includes baseline epidemiological information, socio-economic and cultural characteristics, geographical and environmental features, ethical considerations, policy and legal features, financial, political, and historical factors. On the other hand, the enabling environment encompasses institutional structures including roles and responsibilities, participation and capacity building of stakeholders and other actors and the presence of supportive legal and policy frameworks including their implementation and enforcement [10, 11]. These factors can affect the delivery and evaluation of interventions contributing to the achievement or failure to achieve, the expected impact. Consideration of the context early enough during programme inception and planning will create space for recognition of inherent challenges and devising of ways of adapting to forestall project failures.

It is widely accepted that the control and eventual elimination of *T*. *solium* infection will require a One Health approach. This approach is strongly advocated for by many including the FAO/WHO/OIE tripartite guide[12, 13]. The Tripartite Guide to Addressing Zoonoses in Countries provides a practical manual to guide the “prevention, preparedness, detection and response to zoonotic threats at the animal-human-environment interface” using multi-sectoral principles[13]. One Health approaches are inherently trans-disciplinary, integrating knowledge both from different science disciplines and stakeholder communities to facilitate collaboration [14, 15] with a strong focus on the environmental, ecological, social and economic factors in the control and mitigation of human and animal health challenges [16].

Several frameworks exist to guide the design, implementation, and evaluation of control strategies against zoonoses in consideration of One Health principles. Ruegg et al. [17] have put forward a comprehensive blue print on evaluation of the ‘One Health-ness’ of such projects and programs, this framework has since been used by Paternoster et al. [18] to evaluate the degree of One Health implementation in the integrated surveillance of West Nile virus in Italy. Bardosh [19] has proposed a socio-anthropological framework and critically discussed its key domains by assessing three case studies involving the scale up of interventions against rabies in Tanzania, trypanosomiasis in Uganda, and achievement of total sanitation in Zambia. Braae et al. [20] have put forward the only current guide specifically for *T*. *solium* interventions, that is a step wise approach to guide the design and implementation of interventions against *Taenia solium*.

To the best of our knowledge this is the first study to critically evaluate how the contextual factors have been considered within *T*. *solium* control programmes. The present study considers hitherto understudied aspects of the context and the enabling environment which may have contributed to the success or failure in the implementation of *T*. *solium* control interventions. We identify and discuss the important factors to consider and the potential challenges which might arise if the context is not factored in project planning, implementation and evaluation illustrated by examples in the published literature and the personal experience of researchers involved in such programmes. The description and explicit reporting of these contextual factors can assist in the design, implementation and evaluation of *T*. *solium* control programmes.

## Materials and methods

### Ethics statement

Ethical approval was obtained from International Livestock Research Institute’s (ILRI) Institutional Research Ethics Committee (ILRI-IREC), reference number ILRI-IREC 2019–20. An informed consent form was sent as an attachment on the email inviting the key informants for the interview. Informed consent was obtained from the respondent by signing an electronic copy of the consent form or responding positively on the invitation for interview. Before the start of the interview session verbal consent was sought for recording of the discussion.

### Study design and data collection

#### The research questions

The overall research question was: “What aspects of the context appear to support the success of *T*. *solium* interventions?” Specific questions included: (i) which contextual factors influenced the design and planning of the intervention? (ii) which aspects of context influenced the implementation and evaluation of the interventions? and (iii) what steps did the projects take to address the different contextual factors and with what results?

### Sources of data

#### Systematic literature review

**Fig 1:**
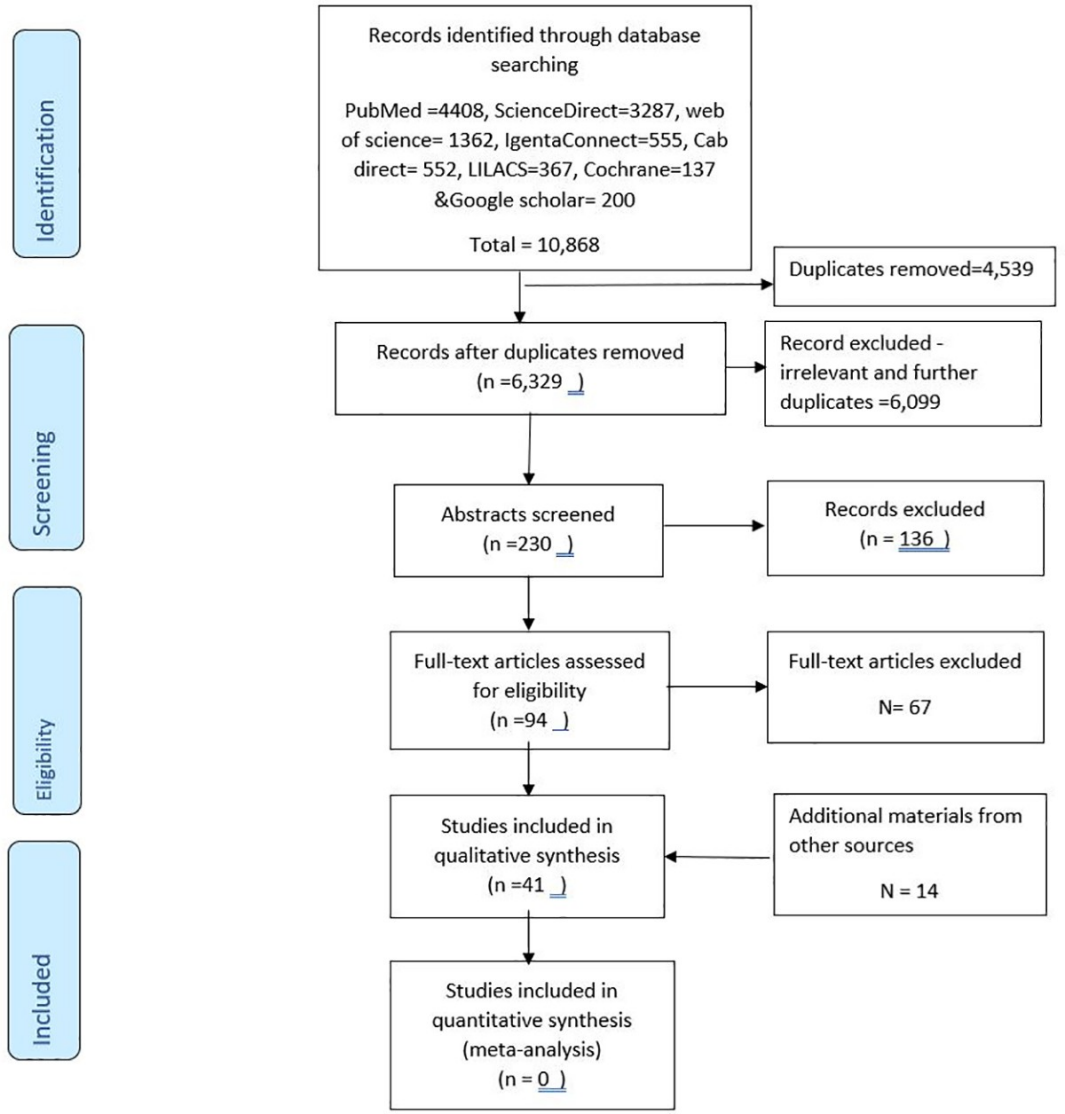
PRISMA flow chart of the selection process in the systematic literature review of contextual factors for *T*. *solium* control.

A systematic literature review on field-based interventions against *T*. *solium* published between January 1950 and May 2019 was conducted to identify what aspects, if any, of the context and enabling environment were reported in the literature. Studies from any country published in English were eligible for inclusion. The SLR was conducted following the PRISMA guidelines for conducting and reporting systematic reviews. The PRISMA flow chart is shown in Fig 1 and the PRISMA reporting checklist can be found in supporting material S1 Checklist. A protocol was pre-registered at the International prospective register of systematic reviews (PROSPERO) number CRD42019138107.

### Search methods, inclusion, and exclusion criteria

The search was conducted in the following electronic databases: PubMed, CAB direct, World of Science, African Journals Online (AJOL), IngentaConnect, Google Scholar and LILACS (This contains most important and comprehensive index of scientific and technical literature of South America and the Caribbean) using the Boolean operators “AND” and “OR” to combine the relevant search terms depending on the specification of the particular electronic database. The search algorithm used in PubMed was as follows;- ("*Taenia solium*" OR "*T*. *solium*" OR Cysticerc* OR Taeni*) AND (control OR elimination OR eradication OR integrated OR Random OR “Clinical trials” OR Challenge OR efficacy OR Praziquantel OR niclosamide OR albendazole OR "mass drug administration" OR "TSOL18" OR vaccination OR oxfendazole OR education OR latrines OR sanitation OR husbandry OR "pig housing" OR confinement OR "meat inspection" OR "hand washing" OR integration OR Biosecurity OR “community sanitation” OR “community hygiene programs”). The search terms were adapted to the other online databases depending on their specifications.

The following exclusion criteria were used: studies not relating to humans or pigs, studies not relating to Neglected Tropical Diseases (NTDs); studies on aspects of NTDs which do not discuss issues relevant to *T*. *solium* control; studies on epilepsy NOT related to NCC; studies on other parasites except soil transmitted helminths; papers relating to clinical symptoms; experimental studies not community-based; diagnoses and treatment of NCC including case studies; purely epidemiological studies on *T*. *solium*, papers on diagnoses of *T*. *solium* cysticercosis/taeniasis (including diagnostic imaging) and papers on aspects of basic sciences (immunology/molecular biology/physiology). For articles published in a language other than English, the abstracts were screened first and if they met the inclusion criteria, the English version of the full article was searched and if not found the articles was excluded. Additional articles were identified by going through the list of bibliographies in selected articles. The risk of bias of individual studies was assessed subjectively by the first author by marking the studies as either low, medium and high risk of bias based on availability of a description of sound methodology with regard to selection of subjects, data analysis and clear and complete reporting of the results.

The key elements of the review question are as simplified by the PICOT acronym below.

- Population: humans or pigs
- Intervention: Drugs for prevention and treatment (Praziquantel, niclosamide, albendazole; mass drug administration of either albendazole or praziquantel or both, TSOL18, vaccination, oxfendazole), education on use of latrines, sanitation, hand washing, pig husbandry, pig housing biosecurity; meat inspection, and “integrated community sanitation” or “community hygiene programs”
- Control: Non-treated, local/experimental study population or none
- Outcome: Efficacy, side effects, acceptance, costs, risk factors, change in knowledge, attitude and practices, prevalence, features of study area at implementation, Conceptual framework/impact pathway, stakeholders involved, challenges encountered.
- Time frame: Time limits–manuscripts published between Jan 1950 and May 2019.

Data from the manuscripts were extracted by the lead author into a Microsoft Excel spreadsheet developed and discussed with the other authors. Data was extracted on the type of the intervention implemented, country of intervention, the indicators monitored, and changes identified attributable to the intervention. Further aspects of the context were extracted, guided by the analytical framework outlined below.

### Key informant interviews

Key informant interviews were then conducted with key researchers from the studies identified through the SLR to further discuss their experience of how context and enabling environment influenced intervention design, implementation, and outcomes for *T*. *solium* control.

A key informant interview discussion guide was developed by the lead author and discussed among the co-authors. The KII discussion guide focused on the planning phase of the intervention, the type and roles of stakeholders involved, the supporting policy environment and context in which the interventions were implemented, how the implementation phase was carried out, a description of the evaluation stage and finally an outline of any challenges encountered.

Key informants invited for interview were either the principal investigator or a supporting researcher. Six respondents were female and five were male working in various capacities in different institutions across the world at the time of interview. Further demographic details on the key informants have been deliberately excluded from this publication to protect the anonymity of the participants since they could be identified by their peers in the field. All interviews were conducted in English and transcribed verbatim.

### Analytical framework

The current study, builds from the socio-anthropological framework for NTD control, [19], the step-wise approach for control of *T*. *solium* [20] and from other literature[9–11], [21]. Craig and colleagues [9] described the contextual factors to consider in population health intervention research which was adapted to highlight and discuss aspects relevant to *T*. *solium* interventions. The contextual factors and their relationship with the interventions are described in Table 1 and were used to guide a mixed inductive and deductive thematic analysis facilitated by NVivo software version 12 [22]. The coding involved the identification of themes sitting within the adapted Craig et al., [9] framework through close line by line coding after thorough examination of the KII transcripts [23]. New themes were identified and discussed with the co-authors before inclusion into the coding frame. New themes were identified until data saturation appeared to be reached due to no new themes emerging. Data extracts (in italics) are included in this article and are identified with the data source (Key informant ID).

**Table 1:** A synthesis of the contextual factors for *Taenia solium* control interventions adapted from Craig et al. [9].

Contextual factor	Description	Examples and applications to the case of *Taenia solium* interventions
Epidemiological factors	Baseline incidence, prevalence, and distribution of the health problem of interest and its determinants in the target population	Baseline prevalence and incidence of PCC, taeniosis and NCC as driven by the underlying biological and socio-economic risk factors, the measure of the outcomes and its reliability, diagnostics methods used.
Socioeconomic factors	Distribution of social and economic resources among communities or populations affected by the intervention, water health and sanitation coverage and education levels	The motivation for rearing pigs, whether farming is subsistence or for income generation; current husbandry practices, including who makes decisions about how pigs should be reared and who provides the labour for pig rearing; level of knowledge and willingness/ability to change practices, including adopting the intervention technologies, income distribution among farmers, access to land or other resources, language, ethnicity, etc. that could affect interventions, other economic activities within the target area
Cultural factors	Beliefs, attitudes and practices among farmers, policymakers, practitioners and those targeted by the intervention, cultural factors relating to pork consumption.	Beliefs, attitudes and practices surrounding pig rearing (pigs are supposed to be “natural cleaners/sanitation policemen” by eating human faeces), pork consumption (e.g. eating raw pork), and *Taenia solium* infection particularly NCC, (cultural norms and taboos around use of toilet), Local taboos/stigma on open defecation, knowledge of the disease and its impacts on their health and livelihood
Geographical and environmental factors	Features of the immediate or more distal (e.g. regional or national) physical environment, either natural or built.	Physical environment including natural and built environment, seasonal variation, access roads, target community location in relation to physical features like mountains, presence of rivers and ponds/lakes–potential for human effluent to contaminate and source of surface drinking water, use of river water for irrigation
Service and organizational	Characteristics, such as readiness to change and motivation, of the individuals delivering the intervention, the organizations in which they work and the wider service environment in which those organizations operate. Co-interventions that target the same risk factors, behaviour or outcomes within the same population as the intervention of interest	Ministry of health and Ministry of livestock, local provincial administration, One Health units, local and international non-governmental organizations (NGOs), willingness of ministries of health and livestock to support interventions, capacity and motivation of local government staff involved in project activities, willingness of local institutions including universities and NGOs to collaborate in the control of *T*. *solium*, competition for time allocation between ministry and project activities. Existence of national deworming programs in the community or schools.
Ethical considerations	The extent to which implementers and recipients understand and agree about the benefits and harms of the intervention and can provide informed consent of exposure to the intervention and participation in associated research	Target population’s common understanding about the benefits and harms from the intervention, capacity to make informed decision and give consent to participate, community empowerment to give consent on their own behalf and on behalf of their dependents especially for therapeutic interventions.
Policy, strategies and legal guidelines	The wider policy framework within which a specific intervention is embedded	*T*. *solium* control should be embedded in country’s livestock disease control policies. Enforcement of some guidelines (for example meat inspection guidelines, local laws on pig husbandry)
Political	Distribution of power among stakeholders and others with an interest in promoting or obstructing the optimum design or implementation of the intervention	Power dynamics among stakeholders, structure of government. Political structures including influence and power of local administrators, interest of the local political leadership in the intervention
Historical	Continuing influence of past conditions, socio-political relationships, policies and legal frameworks	Influence of past involvement of target community in disease control interventions, positive or negative experiences with certain organizations
Financial	Sources and mechanisms of funding for the intervention and the wider payment, reward, incentive or charging structures in which they are embedded	Sources and mechanisms for funding for the intervention, costs versus the benefits, expected budget allocation by ministry of health and ministry of livestock, stability of funding during project implementation period.

## Results and discussion

### Systematic literature review descriptive results

**Fig 2:**
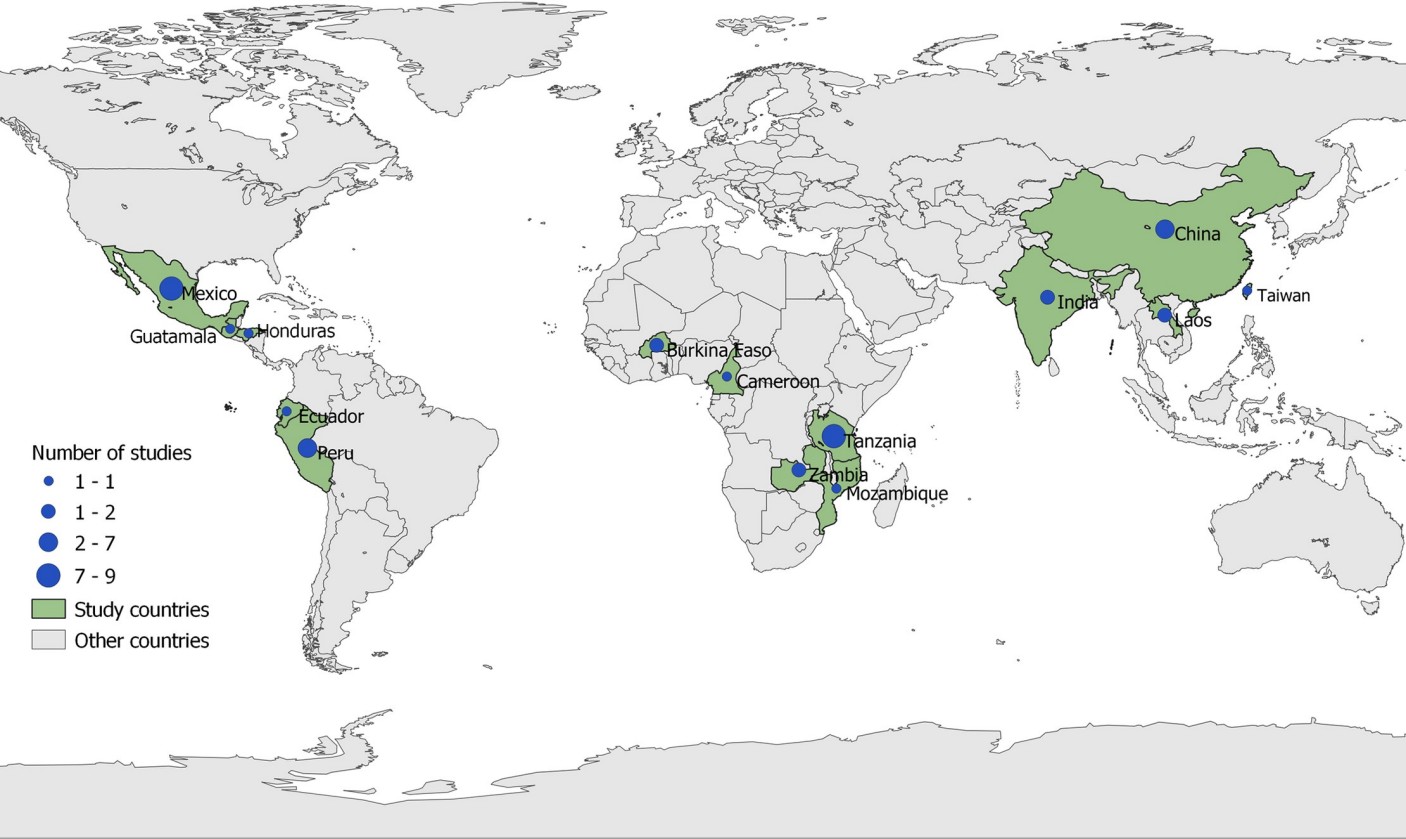
Distribution of studies included in the SLR on contextual factors for *T*. *solium* control (Map: Fredrick Otieno, ILRI). **Base map Link:** **http://www.geoboundaries.org**.

The search identified 10,868 abstracts of which 41 were retained after removing duplicates and screening under various inclusion and exclusion criteria (Fig 1: PRISMA flow chart). The studies included were from 3 different continents: Africa 15, Latin America 18, and Asia 8 studies (Fig 2). Most studies reported results of control strategies targeting one of the three diseases: PCC (17) and taeniosis (8) or various combinations of the diseases (16). No study focused entirely on NCC prevalence or incidence in the short term. There was a high level of heterogeneity between the different studies and therefore it is impossible to carry out a meta-analysis to achieve a single estimate of the effect of the interventions. This is because the different studies used different outcome measures and diagnostic techniques. The identified studies also had paucity of information on the enabling environment and contextual features underpinning the design, implementation, and evaluation of the interventions. However, more information was collected through the key informant interviews. In total 33 studies were scored as being of medium quality and 8 studies of good quality based on subjective assessment by one author.

### Key informants identified

Of the forty-one journal articles identified for inclusion to the qualitative analysis, five listed the same corresponding authors as another article. Eleven out of the 36 articles had no listed email address for the corresponding author and a further 2 had email addresses which did not work, and no alternative emails could be found. In total 23 corresponding authors were identified for the interview, of which eleven authors agreed to be contacted for a key informant interview representing a 48% response rate.

### Describing the contextual factors and enabling environment supporting control of *T*. *solium*

The manuscripts identified through the SLR had a paucity of data on the contextual factors identified through the modified Craig *et al*.,[9] framework. We were able, however, to identify some contextual factors from the methodology and discussion sections which were further explored during the key informant interviews (S1–S3 Tables) containing summary of the studies included in the SLR process. The analysis of contextual factors presented here is organized according to the category of study. This was because the influence of context and enabling environment may vary depending on the study type and scale of implementation. The categories are those which focused on testing the efficacy of interventions under controlled conditions, those which tested effectiveness of control intervention under “real-world” conditions, and finally those which focused on implementation or scale-up of interventions.

In the analysis we show how the different contextual factors were considered by the implementers of the projects reported in the primary studies and how the implementers adapted to changes in context during implementation.

### Contextual factors in efficacy studies

Generally, efficacy studies are performed in an experimental setting or under ideal conditions where most of the parameters are controlled. Their design increases the chances of detecting effect if it exists but does not consider wider contextual factors that may influence an intervention’s effect at scale. The studies identified were mostly field based with limited geographical coverage.

Ten studies focused on testing the efficacy of different drugs and education programmes. These included administration of albendazole in humans which showed limited success in treatment of taeniasis [24, 25], oxfendazole in pigs [26], vaccination against PCC using a variety of vaccine candidates [27–31] health education [32] and use of pumpkin seed (*Cucurbita moschata* Duch) and areca extract (*Areca catechu*) [33]. These studies aimed at proof of concept and most of those testing drugs were conducted in the late 1980s and early 1990s a time when efforts to identify a suitable cestodocidal drug for taeniosis were ongoing. Few contextual factors were considered in these studies and those that did were strongly associated with the logistics of trial delivery and interpretation. Four of the contextual factors were identified as being considered in efficacy studies and a summary of these can be found in Table 2.

**Table 2:** Summary of contextual factors analysis for efficacy studies.

*Contextual factor*	*Specific examples from the studies*	*References and country of focus*
Epidemiological factors	○ Challenges in evaluation including loss to follow-up ○ Reduction in samples sizes due to loss to follow up could have reduced the statistical power of the evaluations ○ Lack of cooperation from study participants.	[24, 25] in Honduras and Taiwan respectively [24, 29]both in Mexico
Social and economic factors	○ Commercially oriented farmers were more supportive of the control interventions in areas where pigs are an important source of income. ○ Farmers may not retain pigs recruited for study in absence of an alternative or incentive/compensation.	[30] in Mexico
Cultural factors	○ Importance of traditional medicine among the community supported uptake of the intervention ○ Gender roles affected the participation of women in training sessions.	[32, 33] in Tanzania and China respectively.
Service and organization factors	○ Extensive sensitization of the local community and involvement of various stakeholders could translate to smooth delivery of the intervention	[31] in Peru

#### Epidemiological factors

For de Kaminsky [24] and Chung [25] who tested the efficacy of albendazole in treating taeniosis in Honduras and Taiwan respectively, little is reported on the contextual environment they operated in and the challenges reported are mostly technological including the unreliability of the diagnostic techniques used. de Kaminsky [24] cited lack of cooperation from the study participants especially in stool sampling and recovery of strobila as the main challenge during evaluation, highlighting the need to fully engage communities in research to ensure the indicators selected may be accurately measured.

For all the studies targeting pigs for treatment or vaccination the major challenge cited was loss to follow up due to several reasons including sale of the pigs, slaughter of the pigs for home consumption or death of the pigs and reproduction state where pregnant sows were excluded. Reduction in sample size leads to a reduced statistical power to detect an effect, potentially impacting the overall findings of the study. Farmers in most cases rear pigs for subsistence and projects having longer follow up periods should consider providing incentives to farmers not to sell the pigs under study. In their study in Cuentepec Mexico, Sciutto et al. [29] reported a loss to follow-up of 215 pigs (56%) where the pigs were sold or were missing. Huerta et al., [28] also reported that 18 vaccinated and 20 control pigs died of causes unrelated to vaccination or to cysticercosis and were excluded from the study. Morales [30] worked in a community in Mexico where 70% of the farmers kept the pigs for commercial purposes, lack of an alternative for these farmers when their pigs were recruited for the study may have led to high loss to follow-up since farmers depended on the pigs for income and would have to sell them.

#### Socio-economic factors

Although suitable trial designs will have been chosen for their methodological rigour, socio-economic context may necessitate opting for modified designs. For example, in Steinmann et al.[34], on evaluating the efficacy of single-dose and triple-dose albendazole and mebendazole against soil-transmitted helminths and *Taenia* spp., the costs associated with double-blind trial implementation were deemed inappropriately high and an open label trial design with the outcome assessors blinded to ensure validity was chosen as a more appropriate design for the context.

#### Cultural factors

Li and colleagues [33] tested the use of pumpkin seed and areca extract to treat taeniasis and found that it was effective in expelling whole tapeworms but with some transient and well tolerated side effects in 46.3% of the study subjects. Cultural context played a big role in this study where the importance of traditional medicines over modern medicine in Chinese communities supported uptake of the intervention.

Uptake of health education interventions is often influenced by gender and education level; consequently, choice of participants and scheduling of the training sessions, should consider them to ensure representativeness and avoid bias. However, though the study by Ertel et al.[32], was not explicit on these contextual factors, the results indicated that neither gender nor educational level influenced knowledge uptake showing that design may overcome cultural factors that would otherwise constrain uptake.

#### Service and organization factors

Jayashi et al. [31] worked with district level partners and a national university in the implementation of a study in Peru to test a combination of two recombinant antigen vaccine TSOL16 and TSOL18. Sensitization of the target community was achieved through individual household visits resulting in community support of the intervention.

*“In Peru everybody recognizes The National University of San Marcos so that was useful*. *So if you say you are coming from The National University of San Marcos they all know and that is a good point [in gaining entry to the community] …[because the university is]well recognized”* KII06.

In the study by Li et al. [33], appropriate sensitization and the participation of the local County Centres for Disease Control (CDC) supported the success of the project.

### Contextual factors in effectiveness studies

Effectiveness studies are performed under “real world conditions” and those identified for *T*. *solium* control were predominately community based, although some of the characteristics overlap with those of efficacy studies [35]. Twenty-six studies focused on testing the effectiveness of various intervention technologies or a combination of interventions. The impact pathways of these research projects were to demonstrate effectiveness in the medium term and demonstrate their use in programmatic settings to achieve sustained impact. These studies included anti-parasitic treatment of people, pig replacement and mass screening [36]. Implementation of health education [37–43] and treatment of humans with niclosamide [44, 45], praziquantel and niclosamide [46]. MDA in humans with albendazole [34, 47–49], praziquantel and niclosamide [41], and MDA with praziquantel alone [50–53]. The treatment of pigs with oxfendazole [54, 55] MDA with praziquantel in humans combined with dosing of pigs with oxfendazole [56], and the vaccination of pigs with TSOL18 vaccine combined with dosing with oxfendazole [57]. The contextual factors are discussed below and summarized in Table 3.

**Table 3:** Summary of contextual factors analysis for effectiveness studies.

*Contextual factor*	*Specific examples from the studies*	*References and country of focus*
Epidemiological factors	○ Baseline prevalence of the disease could modify the goal of the project and measures of effect ○ Loss to follow up was a major challenge	[49, 58] in Lao PDR
Socio-economic factors	○ Baseline knowledge can affect delivery and evaluation ○ motivation to rear pigs and importance of pigs in the community can influence adoption ○ Language for delivery of especially education messages should considered. ○ Baseline anthropological studies to understand the socioeconomic and cultural characteristics of target community are important	[59] in Zambia, [58] in Lao PDR
Cultural factors	○ Baseline anthropological data on beliefs, attitudes and practises which may help maintain *T*. *solium* transmission within a community and which may be hard to change can help understand and mitigate risks to success	[49, 58] in Lao PDR
Geographical and environmental factors	○ Natural and built environment can influence the implementation; for example, poor accessibility of study sites due to lack of roads, challenges in evaluation due to lack of sample handling and storage facilities ○ Seasonality of rainfall and cropping season may affect participation in interventions and may also lead to loss to follow-up.	[40, 48, 56, 57] in Tanzania, Lao PDR, Peru and Cameroon
Service and organizational factors	○ Local capacities of staff and institutions can affect the delivery of intervention ○ Stakeholder involvement and sensitization of local communities on the activities and benefits of the project can influence the support and adoption of the interventions ○ Incentives to ministry staff involvement in implementation are important.	[40, 50, 58, 60] in Tanzania, Lao PDR and Ecuador
Policy and strategies on *T*. *solium* control	○ The approval and acceptability of drug administration interventions can be influenced by country laws on licensing of the drugs to be tested ○ Integration of *T*. *solium* control with existing disease control programs can have synergistic effect and opportunities exist to embed *T*. *solium* control within other national disease control efforts.	[49, 61] In Lao PDR and Tanzania
Historical factors	○ Past involvement of target community and their experiences about other projects can shape their participation in future projects—previously beneficial projects may encourage participation in other projects	[36, 48] in Lao PDR and Peru

#### Epidemiological factors

Establishing and understanding the epidemiological context of the study area is important in guiding the process of setting goals, choosing the intervention to implement and deciding which methods and diagnostics techniques to use to measure impact. For example, in Laos PDR, where taeniasis was hyperendemic, the goal of the intervention was to lower the incidence rates with the overall goal of reducing the burden of neurocysticercosis [49].

*“……we basically did a therapeutic intervention in both humans and pigs and that was the first decision; from all the control intervention which one is the most appropriate for that particular context”* KII04.

The process of designing interventions at scale including the calculation of sample-size, unit of randomisation may be enhanced using transmission models. Several models specifically for *T*. *solium* transmission have been developed which include EPICYST model [62], CystiSim [63], decision tree model [64] and the Reed frost stochastic model [65] as comprehensively reviewed by Dixon et al. [66].

As was the case with efficacy studies, loss of participants (both porcine and human) to follow up has been cited as a major challenge during evaluation by many of the primary studies due to the reduction in sample size. Failure to have plans in place to adjust for the changes in sample size during evaluation could put the validity of the results into question impairing the effect or impact of the intervention. In the study by Ngowi and others [38] in Tanzania on the financial efficiency of health education, seasonal availability of feeds led to 52% drop out of the baseline farmers. Timing for baseline surveys should be done to adjust for fluctuations in prevalence due to seasonal confinement of pigs following the cropping seasons. This will ensure evaluations are based on the correct base prevalence as was recommended by [55] and [57].Additionally, outbreaks of African Swine fever in the neighbouring regions may have discouraged participation by the pig farmers with negative implications on the measurement of effect.

Assana and others [57] in a field trial of the TSOL18 vaccine in Cameroon compensated the farmers at the rate of 12 Euro monthly for hosting the animals during the trial and this could have averted possible high loss to follow up. In their study 28 pigs (11.6%) out of 240 pigs (10 from the vaccinated group and 18 control) were unavailable during the evaluation. To remedy the challenge of loss to follow up statistical analyses have been used for adjustment to ensure representation as was done in [23, 30] and [57].

#### Socio-economic factors

Social and economic factors have far reaching effects on the delivery and sustainability of intervention programs. The motivation for rearing pigs and the relative importance of pigs in the community can influence their participation and adoption of *T*. *solium* control interventions. In Peru, O’Neal and colleagues [45], observed that the community had a lot of interest in the control of *T*. *solium* possibly because they kept pigs for income generation. Steinmann and colleagues [47] noted the high cost of constructing a toilet ($300) in a village in China which could have affected the sustainability if farmers cannot afford after the end of the project. In Tanzania, Kabululu and others [55], note that the use of local construction materials like tree poles, gravel and timber was encouraged to construct pig pens. The project ensured farmers provided free labour during construction to encourage participation and enhance learning. Interventions targeting building of sanitation or animal husbandry infrastructural facilities should emphasize the use of locally available construction materials in order to ensure sustainability of the program.

Findings relating to economic barriers to control, strengthen the call for conducting ex-ante and ex-post economic analysis. Cost-benefit or Cost-effectiveness analysis can be used to identify the costs and benefits (financial, health or societal) accruing to different stakeholders. Narrod et al. [67] proposed the modified risk analysis framework which utilizes stakeholder engagement throughout the process to help assess the societal cost of zoonotic diseases to all sectors involved in control. Examples exist on how to understand the societal costs and benefits of intervention or surveillance of zoonotic diseases which allows for appropriate cost-sharing scenarios to be considered based upon the benefits accruing to the veterinary or human health sector, or across the public or private space[68].

Immigration of people may pose a threat to the sustainability of the elimination campaigns due to the possibility of re-introduction of infection where people with taeniasis may migrate into diseases free areas as was the case in Tumbes, Peru [36]. Pigs and pork with cysts could also be transported into areas that have achieved eradication. A well-functioning surveillance system operating at the community level has, however, been recognized as a potential solution to this threat. Furthermore, economic policies (e.g. bilateral laws penalizing cystic meat) operating at national level can help drive adoption of *T*.*solium* control interventions and prevent reintroduction of infective material as was noted by Bardosh and colleagues [58].

#### Cultural factors

Cultural factors particularly beliefs, attitudes and practices including religious beliefs may influence the effect of the interventions and they are often deep rooted and hard to understand and change. For example, in Lao PDR, the study village had religious significance of consumption of raw pork during ceremonies throughout the year [49, 58]. These practices may maintain transmission within the community even with *T*. *solium* control interventions being implemented. Cultural belief and taboos especially on toilet use have been shown to be a barrier to adoption of behaviour change messages in studies in China and Zambia [47, 69]

Very few studies carried out an anthropological study prior to implementation of the control programme to understand the sociocultural context and the knowledge attitude and practices of the participants. Okello and colleagues [48] had a medical anthropologist in the team who helped understand the sociocultural and economic characteristics of the target community in Laos PDR. They found that transmission was influenced by social determinants including limited market access, interrelationships between alcohol, ancestral sacrifices and the consumption of raw pork, seasonal variations and poor latrine coverage [58]. In a later study, the same author also described the need to understand the local community while discussing the effectiveness of neglected tropical disease interventions under the social difference and community agency domain of the anthropological framework to improve effectiveness of NTD interventions [19].

*“we did some social research because we had a medical anthropologist in the team who did a lot of the background sort of focus group discussion with people [to understand them and get their inputs]”* KII04.

Rapid assessment techniques can be used to understand the social determinants which may affect the success of *T*. *solium* control interventions [58, 70]. These preliminary studies will also help in making the right choice of the intervention to implement which fits in to the local sociocultural settings. Ngowi et al. [40] noted that few women attended the training session of the health education intervention despite being the ones who rear pigs due to their commitment in their farms at the time of the training sessions raising question of reach of the education campaign due to differential gender roles.

Keilbach et al. [46] in a study in a Mexican village noted that high illiteracy levels made it hard for people to give up traditional practices which exacerbate *T*. *solium* transmission. This may require interventions geared towards behavioural change. Extensive behavioural and anthropological studies to guide the design of health education intervention to prevent failure to change behaviour as was the case in [37–39] may be necessary. Although, Hobbs et al [59] reported that delivery of an educational intervention required only a laptop, projector and small generator, this technology may act as impediment to uptake in some areas where computer skills may be lacking even among government officials.

Language can also impact the delivery of health education intervention and evaluations if data collection instruments and training materials are not understood by the respondents. In the preliminary evaluation of the computer-based *T*. *solium* education program ‘*The vicious worm’* by Hobbs and others [59], participants failed to understand the connection between ingestion of invisible tapeworm eggs and human NCC due to use of complex language. However, the study used a unique approach where the epidemiologic and economic evidence of the impact of the parasite were discussed by the workshop participants and provided an opportunity to re-examine the life cycle and leading to an appreciation of its impact and endemicity. Large scale uptake and sustainability may be compromised if knowledge does not diffuse to non-participants due to use of technical language. Use of Swahili language—the widely spoken and national language of Tanzania helped in the delivery of the health education interventions implemented as reported in [37, 39] The pork tape worm life cycle is not easy to understand, even if simplified and non-technical language is used, and general visualization through pictures can also help deliver the message to communities with low literacy levels.

#### Geographical and environmental factors

Geographical and environmental factors also play a role in supporting or influencing the success of *T*. *solium* interventions by affecting coverage and uptake. In Ngowi et al. [40], few women attended the training sessions raising questions of reach because they were busy in the farms during the intervention period as it was planting season. These authors acknowledged that most pigs were reared by women and their inability to participate, could have adversely impacted the effect of the health education intervention. Aspects of the natural environment may limit access to some study sites in places where road infrastructure is not well developed or creates a conducive environment for transmission through use of un-boiled drinking water from natural sources. For example, in Lao PDR, some study villages were unreachable for over 6 months during the rainy season as reported in [49] and two of the 11 key informant respondents. Garcia et al.[56] indicated that the choice of the study site was influenced by accessibility by road and the proximity of the site to the city where specimen handling, centrifugation and storage facilities were available. To adapt to this challenge, implementers may decide to have their own sample handling facilities or plan the field visits to fall in times of the year when the roads are passable as was done by Ash and colleagues [49] and by Okello et al.[48]. At times the purposive choice of study site may introduce bias, but it could be the only possible way out to allow implementation with limited resources and time.

#### Service and organizational factors

Service and organizational context including local capacity in terms of availability of qualified staff to carry out the project activities was also considered vital. All the KII respondents reported that working with the local ministry officials was mandatory for successful implementation. However, the officers needed to be sensitized on project goals, objectives and activities as part of building capacity for the interventions. This was reported by 9 out the 11 KIIs and 4 articles presented in detail how they involved local staff from the relevant ministries[34, 50, 52, 56]. The involvement of local ministry staff is a necessity to ensure cooperation and participation. However, there is need to coordinate project activities and other ministry activities as to avoid conflict between them, as was observed by Ngowi et al. [39].

*“The medical guys we worked with were really smart and some were doing their masters online and were really willing and clever*. *We didn’t have to bring anybody from outside*, *the project team went there and we conducted training to their medical staff and animal health workers to train them on the specific of the sampling and we were very confident that they were able to do everything we required… So*, *staff capacity was brilliant”* KII11.

It was indicated by key informants that government employees involved in the implementation required incentives which may be of two types–per-diems and non-monetary incentives. Ten (10) out of 11 key informants agreed that donor funding must budget for provision of per-diems and other field allowances to ministry of livestock and ministry of health staff working on the project. This has been the practice in many African countries where the ministries are in most cases under-funded and development projects are donor funded in many instances. This position has been supported by Bardosh [19] who discussed the need for providing incentives to field staff in the effectiveness of neglected tropical disease interventions framework. The non-monetary incentive included building capacity of the local staff. The additional skills acquired through short training and higher levels of training like post graduate studies may help in advancing their careers. Many projects had this component: in form of competitive masters and PhD degree scholarships.

*“We have a formal capacity building in the projects in form masters and PhD students*, *but we also do a lot training*. *So district people were taken care of over the project period which was over 6 years”* KII04.

*‘When we work with them*, *we give them per-diem as per the government rates but when we finish*, *we don’t continue giving them because we will not have the funds*, *so the issue of sustainability comes in [when donor funding run out]”* KII09.

Lack of technical capacity may lead to problems in sample handling and management. Sample labelling and inventory especially for large scale projects can lead to reduced power to detect an effect if some samples are lost affecting evaluation. Ash et al. [49] experienced high number of faecal samples which were unidentified pointing to the need for a well-organized sample recording and tracking especially for large scale interventions.

Infrastructural aspects such as laboratories and cold chain are better considered early during the project inception as reported by 6 out of the 11 KIIs. In the study by Okello et al. [48] in Lao PDR the lack of facilities made it impossible to carryout large scale carcass dissection of pigs as part of the evaluation process. In Peru and Zambia long term funded projects led to the establishment of laboratory and other related research facilities in the study sites which may have had a positive impact on the project. Lack of laboratory facilities and unreliability of the diagnostics techniques has often been cited as a big impediment to the evaluation of *T*. *solium* control interventions [39].

*“In Peru at the field site we have administrative facilities and we have animal kraals and we have a clinic*, *CT scanner*, *a lab- we have all that capacity”* KII02.

*“the project was able to put up a small lab in the area*, *just a simple structure with a power generator”* KII-11

*“What is on the ground*, *what structures are there and I think that should define all that you are going to implement”* KII01.

#### Institutional stakeholder involvement

For *T*. *solium* control interventions, a variety of stakeholders were identified from by the KIIs and primary research articles as being vital for the successful implementation. They included national government ministries and agencies (ministries of livestock and health, research approval commissions, and One Health coordinating unit) if present. At the project site level, local government officials, community leaders, community members including diseases victims and community-based organizations (CBOS) should be represented. Also, local and international NGOs, academic institutions (universities and local schools), and pharmaceutical companies are important. For example, extensive involvement of appropriate local stakeholders in peer learning and planning in Peru led to over 90% of the local people supporting the project[43, 50].

Specifically, involvement of local governments and community leaders was also emphasized in papers and by key informants. These include the district level representatives of the national government and the local community leaders like the mayor and chiefs depending on the local administration organization in the target country. Thizy et al. [60] points out that stakeholder engagement can help in shaping the implementation pathways and should start at project onset and maintained throughout the project life cycle. The most common approach was holding one on one meetings with community members to explain the goals and objectives of the project and address any concerns they had about the project. The selection of stakeholders should reflect the specific nature of problem to be addressed [14] and the context within which implementation is to occur [15].

*“I introduced myself to each community leader in the villages so that was very important just to know where I was [with respect to location with their area of administration] and what I was doing”* KII06.

*“As a hierarchical society we made sure to get the village headmen and area chiefs on board because I think we were able to leverage that sort of hierarchical structure and if you convince the area chief on the importance of the intervention they actually do have a lot of weight in getting people to adhere to those”* KII11.

In nearly all the studies the ministry of livestock, particularly the department of veterinary services was involved at the implementation stage where the department provided officers to help in carrying out project activities. This could be attributed to the fact that majority of the interventions–even those focused on human host, measure the outcomes at the pig level due to the ease in obtaining samples and availability of diagnostic techniques.

*“We always had the support of the veterinary side so the agricultural side was always on board and the human side are not sold on the idea of One Health I think particularly on zoonotic diseases and this is a barrier to getting the ministry of health on board”* KII10.

The ministry of health was majorly involved when the intervention was targeting humans. For example, the MDA programs where the local health posts and health officers were involved in administering the drugs and managing side effects [47, 49].

The development of a truly ‘One Health’ control program demands for the cooperation and participation by the veterinary, medical and public health sectors and the success of these programmes calls for the attention to the management and coordination of the various components of these control organization as alluded by Murrell and Pawlowski [71]. To truly embrace the One Health concept, gaining buy-in and collaboration from the ministry of health at an early stage, even when only non-human hosts are being targeted for intervention, would be more appropriate. Thus, participatory approaches of including actors from the human health and veterinary sectors as well as local communities and local authorities to facilitate knowledge exchange should be considered to encourage stakeholder engagement [14, 15, 72].

Two out of the 11 key informant respondents cited the involvement of the actual people infected by the disease as helpful in delivery of the intervention. For example, having people who have been affected by either taeniosis or neurocysticercosis as community volunteers can help make the problem feel more real. This could in turn lead to community support for the proposed initiatives.

*“[people can see] …this is a big worm*..*and then of course where you have cysticercosis the people are afraid of the seizures [and] they are afraid of the consequences of cysticercosis*. *if you have cases of people who volunteer to be presented as affected then you can explain the connection between the people they see who have the seizures and the worm*..*this may have a lot of power to convince the people do something[due to fear of consequences]”* KII03.

*“…*.*there are many other people who have just been affected by the disease*, *a family member has cysticercosis*, *the pigs are sick [may be with other diseases] or they[people] have taeniasis and they [may] become quiet motivated to stay engaged to talk to their neighbors [about the problem]*, *I think the trick is in identifying those people and giving them a capacity to work and share what they know”*KII02.

#### Policy and strategies on *T*. *solium* control

Policy, strategies and legal guidelines in a study country can affect the choice and delivery of intervention since they must be embedded within the country laws and guidelines. In Lao PDR the ministry of health had not licensed praziquantel for use in humans and although it is the recommended drug of choice by WHO for treatment of taeniasis. This coupled with the general acceptability of albendazole by local community due to its ease in chewing made it the drug of choice over praziquantel in the MDA intervention implemented by Ash et al.[49]. The policy environment and existing country laws also influence the adherence to ethics through licensing and monitoring to prevent violations. Consideration of ethics and harm to the research participants may also influence the choice of technology to be trialled especially for therapeutic interventions, for instance, Niclosamide may be considered as a substitute for Praziquantel (which can cross the blood-brain barrier and may lead to adverse effects [51] in areas with a high prevalence of NCC [44, 48]

#### Historical factors

Historical factors, for example influence of past involvement of target community in disease control interventions, positive or negative experiences with certain programs can impact delivery of interventions positively or negatively. In Piura province, Peru, O’Neal et al. [45] demonstrated that ring screening for taeniasis could reduce *T*. *solium* transmission and acknowledged the excellent participation of the local community, possibly due to good rapport which has been created overtime due to continued work on *T*. *solium* control in the area. History of having participated in a bigger project funded by the Australian Centre for International Agricultural Research (ACIAR) made the village in Northern Lao PDR to be supportive of *T*. *solium* interventions [49].

#### Integration of *Taenia solium* control with other programmes

Leveraging the integration of *T*. *solium* control interventions with other country programs on control of other NTDs or wider human and animal health issues can also indirectly afford *T*. *solium* control the much-needed government support.

Wide-spread uptake of this approach, however, will require proof that it will be cost effective as has been shown for combined treatment of schistosomiasis and other soil transmitted helminths through school deworming programs [61]. Braae et al.[53] in their study in Tanzania concluded that utilizing existing National deworming infrastructure under the school deworming programmes may be cost effective approach to the control of *T*. *Solium* infections.

The anthelmintic treatment of people against *T*. *solium* and Soil Transmitted Helminths (STH) as well as treatment and prophylaxis of pigs against *T*. solium and Classical Swine Fever (CSF) in Laos, was both effective at reducing prevalence of each target disease and highly cost effective[48, 73]. Similar observations were made by Garcia et al.[56] who combined human and porcine chemotherapy and vaccination against hog cholera. This approach increased the turnout of pigs sampled during the baseline survey. Jayashi et al.[31] used a similar approach where they vaccinated the pigs against (CSF) alongside the TSOL16–TSOL18 vaccine to encourage more participation by the farmers in Peru.

In many countries, a lot remains to be done in terms of restructuring administrative processes in order to maximize the benefits of integration as reported by [74]. The potential of integrating *T*. *solium* control interventions with other programmes especially those focused on soil transmitted helminths and water, sanitation and hygiene (WASH) programs was emphasized by nine out of the 11 key informant interviews.

*“It is important we think of integrating T*. *solium* control *with other problems which are more recognized but are beneficial to Taenia solium also*. *One of the strategies is the MDA undertaken in school using praziquantel which can also take care of taenia…… the other one is the WASH programmes…*.*”* KII09.

*“Integration…*.*it needs studies to prove that it would actually be cost efficient”* KII01.

However, implementers of combined approaches need to be careful to ensure the observed effect is attributable to the intervention under implementation and that there is no confounding with the effect of the other programmes running in the same study area. Sarti et al.[52] tested the effect of MDA with praziquantel on taeniosis and during evaluation they noted the increase in toilet coverage from 31% during baseline to 64%. This change was not related to any activity undertaken by the intervention under evaluation but probably to WASH initiatives within the study area.

Ngowi et al. [39] implemented a health education intervention in an area where another NGO was issuing heifers to farmers and training them on husbandry practices. Although, the implementers blinded the participants on the objective of the study, spill over effect from the other program may have confounded the results of the evaluation of the health education intervention. Other programmes being implemented within the study site simultaneously with *T*. *solium* control may affect compliance. Ongoing therapeutic programs such as school deworming programs may create concern by parents that their children are being given too many pharmaceuticals, a scenario faced by [47–49]. Proper coordination and information sharing across health programmes may allow a synergistic effect between MDA programmes and positively influence uptake as well as addressing some of the barriers to implementation such as conflict between the goals of different stakeholders [15].

### Contextual factors in scale up or implementation research studies

Implementation studies consider the application of research findings into practice. They must emphasize partnerships between community members, implementers, researchers, and policy makers and by definition need to understand the enabling environment [75].

Three studies identified were classified implementation research projects which aimed to achieve and sustain long term impact. They included MDA with praziquantel under the National Schistosomiasis Control Programme (NSCP) in Tanzania [61], evaluation of the Community Led Total Sanitation (CLTS) in Zambia [69] and the community based education programme developed using the PROCEDE-PROCEED model implemented in Burkina Faso [76]. The three studies had substantial government support in terms of buy-in and policy orientation which ensured active involvement of the local levels of government which has been identified through the key informant interviews as a major constraint to successful implementation of the interventions. The contextual factors are discussed below and summarized in Table 4.

**Table 4:** Summary of contextual factors analysis for scale-up studies.

*Contextual factor*	*Specific examples from the studies*	*References and countries of focus*
Epidemiological factors	○ Problems with compliance have been observed for collection of faecal and blood samples. ○ Reduction in compliance over the lifetime of the project leads to reduced power to measure effect size. ○ Enhanced compliance for collection of faecal samples has been achieved by having a system to discretely submit the faecal samples.	[61, 69, 76]
Social and economic factors	○ Differential participation of men in baseline and end line surveys due to commitments in farms may have affected gendered analysis of the effects of the intervention. ○ There was poor knowledge of cysticercosis in area—prior knowledge of this could have led to redesigning of the intervention to include health education	[69] in Zambia; [76]
Cultural factors	○ Taboos around toilet use among the Chewa people in Zambia ensured open defecation continued even with promotion of toilet use. ○ Some communities may rigid to change and hierarchy in decision making may affect adoption	[69] in Zambia
Geographical and environmental factors	○ Rainfall seasonality affected men attendance to post intervention evaluation meetings because they were busy in the farms. ○ Other economic activities e.g. mining may affect participation in research projects.	[69] in Zambia and [76] in Burkina Faso
Service and organization factors	○ One health aspects of involving all relevant stakeholders were not fulfilled leading to several challenges in the study in Zambia. ○ Holding extensive community meetings which led to support for the project. ○ Finding qualified staff willing to work in field conditions under the low salary was a challenge	[69] and [61] in Tanzania, [76] in Burkina Faso
Policy and strategies on *T*. *solium* control	○ Projects can be embedded within existing National disease control programme	
Financial	○ Stability and sustainability of funding may affect evaluations due to reduced sampling rounds or sample size	[76] in Burkina Faso

The three studies tried to identify the contextual factors which may have affected the implementation of the interventions and tried to adapt to emerging contextual issues during the project life cycle to a greater extent than studies within the previous categories. Bulaya et al.[69] conducted a preliminary evaluation of the effectiveness of the CLTS program in reducing the prevalence of taeniosis and porcine cysticercosis in Eastern Zambia, eight (8) months post intervention, there was no reduction in PCC prevalence. Some of the cited reasons for this included continued open defecation practice possibly due to their cultural practices. Culturally, household male members of different age groups and relationships among the Chewa people of Eastern Zambia, do not share toilets.

Thys et al. [77] specifically investigated the cultural contextual factors impacting the result of this intervention and made a similar observation that men among the Bantus were reluctant to use toilets due to taboos relating to sharing of the toilets with their in-laws and grown up children of opposite gender. Obviously, the planners and implementers of the CLTS programme may not have considered these cultural aspects when initiating the project. Poor knowledge of cysticercosis (only 50% had seen infested pork) observed in this study suggests that it may have been prudent to have implemented a health education intervention alongside the CLTS project or any other intervention as suggested by [39, 78]. Contextual factors can also affect the evaluation process hence compromising the measurement of effect or impact. For example [52], seasonal variation in agricultural activities based on rainfall patterns in the study area, led to more males attending the baseline meetings than the post-intervention meetings. This compromised the gender analysis of the effect of the interventions. Perhaps generating a community map of annual and daily calendar of activities, could have led to a more appropriate timing of meetings to avoid non-participation.

Consideration of climatic and seasonal distribution of rainfall, and hence farm activities during planning for the data collection meetings could have pre-empted the scenarios encountered.

Braae et al.[61] evaluated the effect of repeated MDA plus track and treat of taeniosis cases on the prevalence of taeniosis in Tanzania. The study presented limited information on the various contextual factors they considered in the planning and implementation or which may have posed challenges to the project. However, the authors report that extensive community meetings were held to sensitize the target community about the project. To build trust and ensure the MDA intervention was supported by the target population, a mechanism was put in place to report adverse effects from the anthelmintic to the district health officer or the assigned medical doctor. The project team also reduced embarrassment around the submission of faecal samples by providing containers for sample collection and setting up strategic locations for drop off therefore improving compliance to some degree. Compliance did eventually decline however, reducing the sample size towards the end of the study which made it difficult to measure the impact of the MDA among school age children on *T*. *solium* prevalence, a significant deviation from the set goals of the study.

Lastly, Carabin et al. [76] implemented a cluster-randomised controlled trial to measure the effectiveness of a community based education programme on cumulative incidence and prevalence of human *Taenia solium* cysticercosis in Burkina Faso. The study used implementation research approach with extensive community involvement in the project and a strong partnership with local organizations to successful implement a programme. Water and Sanitation for Africa, a local NGO provided experts who trained facilitators on Participatory Hygiene and Sanitation Transformation (PHAST) model which was the hangar of the project [79].

A comprehensive anthropological study during the project inception, to fully understand the social structures and norms of the target community was not carried out, which resulted in several challenges. The hierarchal nature of one of the communities made that decision are made at the top by the community leaderships and passed on to the general population. This led to poor adoption of the intervention possibly because the population felt left out in the decision-making process. The need to fully engage community level actors and create a collaborative environment between them and the scientific community in order to build trust has previously been highlighted by Hitziger et al.[15]. Additionally, there was refusal to provide blood samples by some of the community members citing the insufficient token of appreciation offered. This may have affected the evaluation of the project and may have been avoided by involving the target participants in deciding on the token to be offered, although this must be balanced against the ethical issues surrounding ‘payment’ of participants for engaging with a research programme which might diminish the sense of autonomy a participant feels in providing informed consent. Other sociocultural factors like the rigidity of the communities to behavioural changes were also cited in this study where it was hard to convince people to adopt use of latrines.

The intervention was delivered in the local language which was felt to result in improved uptake and was reinforced by the close monitoring conducted by the lead investigator and local organization staff ensuring integrity of the implementation. The program identified a challenge in finding qualified staff willing to work in field conditions under the low salary provided by the project. This can be avoided by conducting pre-visits and holding meetings with the relevant ministries so that the expectations of the local staff are considered in the budgeting process. A position supported by Bardosh [19] where he discussed this challenge under the strategies and incentives to staff in the socio-anthropological framework to improve the effectiveness of NTDs intervention.

Geographical context came into play within this programme, when the discovery of gold in the study area during the implementation period, caused people to leave the area to work in the gold mines leading to losses to follow-up. The project adapted to the challenge by replacing the dropouts with another member of the same family and using Bayesian modeling during analysis to increase validity of the results and to avoid bias [76]. Funding stability can also affect the implementation of a project like was reported in this study where budgetary cuts during implementation led to cancellation of one of the planned sampling rounds. The success of many NTD interventions will to a large extent be pegged on continued and sustained funding as was observed by Reed and Mckerrow [80].

## Conclusion

The effect of contextual factors on efficacy, effectiveness and scale-up studies vary and the factors interact to influence the implementation and evaluation of *T*. *solium* control projects. For efficacy studies, context is often limited to the more technical aspects of the programme; classified here as epidemiological factors, since the researchers tend to control the environment under which the studies are implemented.

For field-based effectiveness and implementation studies a wide range of contextual factors were found to be important in the eventual success or failure of interventions. Some of the important contextual factors identified through the literature and key informant interviews were epidemiological factors including baseline and end-line outcomes measures, socioeconomic and cultural characteristics of target participants encompassing local knowledge, attitude and perceptions, geographical and environmental factors, service and organization including stakeholder roles and their engagement. Implementation research projects will need all these contextual factors plus a supportive policy framework to underpin the success of *T*. *solium* control programmes in endemic settings.

We conclude that although it might be challenging to fulfil all the contextual factors discussed, those considering rolling out interventions should consistently evaluate and consider these factors at the planning, implementation and evaluation stages. A full consideration of these factors will also require an inherent consideration of the tenets of One Health principles which we believe are critical for the success of *T*. *solium* control interventions.

### Recommendations

As the research agenda around control of *T*. *solium* progresses from effectiveness studies to implementation research, we recommend a greater focus on identifying, considering and reporting the contextual factors and their potential influence on project impacts and outcomes. The pre-project analysis of the different domains of context at inception and planning should evolve into a written strategy for their mitigation during implementation.

Further studies are needed to generate evidence on the contextual factors operating in the various endemic regions and the expectations of communities regarding the implementation *T*. *solium* control intervention. Evaluation of *T*. *solium* control projects against the One Health principles as proposed in the blueprint by Ruegg et al. [17] and used by Paternoster et al. [18] is also required.

### Limitations of the study

The risk of bias assessment was conducted subjectively by one author and a risk bias graph was not developed which may have in turn introduced bias into the assessment. Assessment of bias of the systematic review was not conducted but the research question and PICOT has been clearly presented. The scope of papers reviewed within the SLR was restricted only to those found using the English Language Syntax and this may have led to exclusion of articles written in other languages other than English. Several manuscripts written in Spanish were returned, representing studies conducted in Latin America and where possible an English translation was found. We acknowledge that there is a body of work from China which was not covered within this study.

## Supporting information

S1 Checklist PRISMA checklist for the systematic literature review of contextual factors for *T*. *solium* control. (DOC)

S1 Table Interventions on efficacy of drugs against *T*. *solium* infections in humans and pigs. (DOCX)

S2 Table Studies focusing on testing effectiveness of control interventions. (DOCX)

S3 Table Studies focusing on implementation research for control interventions or scale-up of interventions. (DOCX)

S1 Data Systematic literature review data extraction file. (XLSX)
