# Supplementary material for: A qualitative assessment of the context and enabling environment for the control of Taenia solium infections in endemic settings
Source: PLoS Negl Trop Dis. 2021 Jun 11;15(6):e0009470. doi: 10.1371/journal.pntd.0009470 (PMC8221787; doi:10.1371/journal.pntd.0009470)
Supplement: S1 Table — (DOCX) [file pntd.0009470.s002.docx]

**S1 Table: Interventions on efficacy of drugs against *T. solium* infections in humans and pigs**

| **Study ID** | **Target population and study site** | **Intervention** | **Features of study area at implementation** | **Conceptual framework/impact pathway** | **Methodology/ study design** | **Outcomes/findings** | **Challenges encountered and opportunities** | **Comments from KII** |
| --- | --- | --- | --- | --- | --- | --- | --- | --- |
| Kaminsky et al., 1991[1] | Honduras; 2% of inhabitants in 15 rural communities; 56 individuals who were found to be infected with taeniasis | Administration of albendazole to people; drug donated by private company; individuals treated with 400mg of albendazole for 3 days and followed at 5 days, 60 and 90 days | The participants were not cooperating; area endemic to T. solium and T. saginata; limited coverage of the population; no information on engagement of stakeholders and sensitization | Aim was to test efficacy of albendazole to treat Taeniasis, evaluation done absence of eggs or proglottides in faeces using KATO cellophane thick smear, scotch tape perianal swab | Baseline conducted and positive people given albendazole at 400mg/kg | 2 % to 0%- all individuals were negative at 60 and 90 days | unreliability of diagnostic method acknowledged, poor recovery of strobila, poor cooperation from study participants; unreality acknowledged -KATO reliability of 80% and combination the two methods 88% reliability; poor collaboration by the study subjects hence poor monitoring of proglottides | Not interviewed |
| Li et al., 2012[2] | Sichuan Province, Yajiang county, China; 117 individuals with suspected taeniasis, 72 were male and 43 were female, follow-up 10 months to 22 months post-treatment | pumpkin seed and areca extract to treat taeniasis | Funded by regional government; worked with Yajiang County Centers for  Disease Control (CDC) involved in sensitization | Evaluation by curative effect- number releasing full worms and those releasing segments measured by Microscopy and CoproPCR | A community-based study, Patients in the study group were given 3 different compounds  in order at 40 min to 1 h intervals, including 120 g of peeled raw  pumpkin seeds, 200 ml areca nut extract for an adult patient, and  magnesium sulfate solution at a dose of 0.5 g/kg body weight. | Following treatment, 40 (88.9%) of 45 cases discharged whole tapeworms, indicating an 88.9% of cure rate, with a 95% credibility interval of 79.7–98.1%, and 2 (4.4%) expelled incomplete strobila, whereas the other 3 cases did not expel worms or proglottids 91 (79.1%) released whole tapeworms 4 (3.5%) expelled incomplete strobila, | The anthelminthic agent in the areca nut proved to be arecoline, The antiparasitic  component in pumpkin seeds was identified as cucurbitine; treatment with traditional Chinese medicine was effective; side effects gastrointestinal upset and dizziness, | Not interviewed |
| Molinari et al., 1997[3] | Guerrero State, Mexico; 12 months for 6 villages and again after another 12 months 4 villages of the 6 vaccinated at 12 months | vaccination with antigenic extract from T. solium metacestode | poor living conditions; no conventional abattoirs, no physicians; Worked with university students | Testing systemic vaccination of pigs; if enough  number of animals are immunized; the parasite might be eliminated over time and integration is possible; the systemic vaccination of pigs concept has been adopted for the new vaccine TSOL18. | 17 villages selected, 2650 inspected by tongue palpation and vaccinated;6 villages selected for subsequent vaccination; third vaccination done in 4 of the 6 villages | at second vaccination among 971 inspected pigs, 12 were  found to be cysticercotic (mean 1.1 _+ 1.01 SD, 1.2%, P < 0.05) and (mean 0.45 + 0.45 SD, 0.46%, P < 0.05). All positive pigs were new cases aged 6 - 8 months. prevalence of porcine cysticercosis | More advanced and effective vaccine has since been developed. | Not interviewed |
| Morales et al., 2008[4] | Morelos, Sierra de Huautla rural community, Mexico; 62 % that is 562 pigs | Vaccination of pigs with S3Pvac; it’s a recombinant M13 phage version of the anti-cysticercosis  tripeptide vaccine | Trained technicians did the tongue inspection; over 25% no latrine; water from well, river and tap water; over 50% use river; 90% of pigs roam free; over 70% kept for sale and the remaining for consumption | Test efficacy evaluated by PCC prevalence by tongue palpation, PCC prevalence by necropsy and number of cysticerci in each carcass | Sixteen rural communities were selected from the 25 in the area. half the members  of each litter were immunized with S3Pvac-Phage vaccine described above and half with placebo (saline) | cysticercosis was diagnosed in 12 pigs  (3.9%) of the vaccinated group and in 29 pigs (13.0%) of the control group, corresponding to a vaccine efficacy of 70%. There now a new vaccine for PCC | 198 in control and 319 in treatment group- consumption, death, natural death or reproduction before necropsy at 27 months 89 lost in control and 110 lost in treatment; the huge costs of synthetic  peptide technology make S3Pvac production unaffordable | Not interviewed |
| Ertel et al., 2017[5] | Mbeya region in Tanzania; 79 participants from health- and agriculture sector- study subjects in group sessions with up to 17 participants per group. | health education using the vicious worm | A total of 79 study subjects were included in the study, hereof58 (73%) employed in the agriculture sector and 21 (27%) in the health sector. Of the study subjects, 17 (22%) were females and62 (78%) were males. Mean age was 36 years (range: 22–59 years, SD = 10). | evaluation of the efficacy of the vicious worm education tool measured by change in KAPs; follow-up study was conducted only after two weeks, measurement of long term effects would be desirable | The study involved the following sessions:(1) a prequestionnaire to assess the study subjects’ baseline knowledge regarding T. solium, (2) individual health education with ‘The Vicious Worm’ for 1½ h, (3) a post questionnaire (identical to the prequestionnaire) | 77% (95% CI: 67.7–86.3) had significantly improved (p < 0.001) their knowledge score immediately after the health education, and for 70% (95% CI: 59.7–80.3) the improvement persisted two weeks after (p = 0.001); The veterinarians had the highest mean knowledge score and the agriculture/livestock diploma students the lowest | The study subjects found the program educative and appealing due to the useful and practical information provided and its depiction of African settings. Some suggested supplement leaflets or similar information material for use in rural areas | Not interviewed |
| Huerta et al., 2001[6] | Huatlatlauca and Tepetzetzintla,  in the state of Puebla, Mexico | Vaccination of pigs with Synthetic peptide vaccine | open defection practiced; local pork meat consumption and extensive domestic pig slaughtering | Efficacy of a chemically synthesized vaccine evaluated by necropsy 10 to 12 months after vaccination. | a controlled field trial with a control group | The results indicate 52.6% efficiency of the vaccine in reducing the number of infected pigs and 97.9% reduction of the total parasite load. Cysts reduced from 15.8 to 7.5%. At 95% level of confidence | Of all these, 18 vaccinated and 20 control pigs died of causes not related to vaccination nor to cysticercosis and were excluded from the  study | Not interviewed |
| Jayashi et al., 2012[7] | Morropon, Piura, Peru; Pairs of pigs (n = 137) comprising one vaccinated and one control animal 19.7% (54/274) loss to follow up | vaccination using a combination  of two recombinant antigens, TSOL16 and TSOL18, | Did not involve a lot of stakeholders except district level leaders and households; no community meetings for sensitization; no plans for scaling up mentioned | Evaluation of the efficacy of the vaccine under natural conditions evaluation by the total number of cysts and the number of viable cysts. Using an effective vaccine in  pigs would remove the source of tapeworm infection in  humans, breaking the parasite’s life cycle and indirectly  eliminating the causative agent of human neurocysticercosis | a pair-matched vaccination trial with 7 months | From the 113 animals vaccinated with the TSOL16–TSOL18 vaccine, 93.8% (106/113) pigs were free of infection. There was a significant 99.7% reduction (Wilcoxon signed-rank test, p < 0.01) in the total number of cysts in the vaccinated group (Total number of cysticerci = 83, mean = 11.9, median = 6, range = 1–54). There was a significant 99.9% (Wilcoxon signed-rank test, p < 0.01) reduction in the number of viable cysts in the vaccinated group (viable cysts = 3, mean = 1.5) versus the animals in the control group (viable cysts = 33,416, mean = 2227.7). | Immunization with the TSOL16–TSOL18 vaccines has the potential  to control T. solium transmission in areas where the disease is endemic | Worked with local contact person who was known in the area |
| Sciutto et al., 2006[8] | Cuentepec in Mexico; 476 piglets - 2 months old were considered but 381 included | Vaccination with synthetic peptide vaccine S3Pvac against PCC in pigs | Free roaming pigs and open defection; local consumption of uninspected  and cysticercotic pork; no mention of stakeholders involved | Baseline conducted by tongue palpation and necropsy of sentinel pigs; single and 2 doses of vaccine; | Randomised control trial | prevalence sentinel pigs, before and after the vaccination study, did not change significantly; a single  vaccination is not enough | Loss to follow-up 215 pigs = 56% sold or missing; low sensitivity of 50% of tongue palpation; labour intensive and not cost effective; time-frame of the vaccination trial made no difference detected | No interview |
| Allan et al., 1997[9] | municipality of Quesada, Santa  Gertrudis and Eltule villages in Guetamala; 1513 individuals (74.9%) coverage | Mass drug administration with niclosamide with magnesium sulphate purgative | Pigs left to roam free; 26% in Santa Gertrudis and 67% in EL Tule open defecated; baseline conducted; niclosamide considered over praziquantel to avoid contraindication with people with NCC; no mention of stakeholders involved | Intervention evaluated by prevalence of taeniasis and PCC by EITB and EPG of *T. solium* eggs after 10 months; reduction of taeniasis will lead to reduction in PCC and HCC; no plans on sustainability or scaling up | A before and after intervention study; niclosamide administered to all consenting individuals | Prevalence changed from 3.5% at baseline and 1% at endline, p<0.0004; PCC declined from 55% before treatment to 7% | MDA consideration; Select an appropriate drug, percentage coverage of the population by treatment, and the appropriate intervention interval if multiple treatments are applied. Optimal retreatment interval not determined; PCC prevalence remained high; human behaviour is a critical factor | Not interviewed |
| Chung et al., 1991[10] | Nanao District,  Ilan County, Taiwan; 66 cases (27 males and 39 females, from 9 to 74 years of age) | Efficacy of Albendazole | Cases identified by questionnaire and passing of proglottides; Private company donated albendazole; no information on stakeholders engaged.  Scaling not recommended because drug is not efficacious | Aim was to evaluate efficacy of albendazole in treating evaluated using cure rate ; no recommendation for scaling because the drug was not effective against taeniasis | A field trial; (1) 400 mg x 1 day, (2) 800 mg x 1 day, (3) 800 mg x 2 days, (4) 800 mg x 3 days, (5) 1200 mg x 2 days and (6) 1200 mg x  3 days. The above six regimens were given to 30, 8,  14, 4, 6 and 4 patients, respectively. re-treatment with atabrine (= mepacrine, quinacrine) (male, 1.2 g; female, 1.0 g) was conducted. | cure rate of 50% of patients who had received  800 mg x 3 days, 1200 mg x 2 days, or 1200 mg x  3 days; in 14.3% of those given 800 mg x 2 days;  and 0% of those who received 400 mg x 1 day or  800 mg x 1 day | Study show albendazole is not effective against taeniasis | Not interviewed |

**References**

1. de Kaminsky RG. Albendazole treatment in human taeniasis. Trans R Soc Trop Med Hyg. 1991;85: 648–650.

2. Li T, Ito A, Chen X, Long C, Okamoto M, Raoul F, et al. Usefulness of pumpkin seeds combined with areca nut extract in community-based treatment of human taeniasis in northwest Sichuan Province, China. Acta Trop. 2012;124: 152–157. doi:10.1016/j.actatropica.2012.08.002

3. Molinari JL, Rodriguez D, Tato P, Soto R, Arechavaleta F, Solano S. Field trial for reducing porcine Taenia solium cysticercosis in Mexico by systematic vaccination of pigs. Vet Parasitol. 1997;69: 55–63.

4. Morales J, Martínez JJ, Manoutcharian K, Hernández M, Fleury A, Gevorkian G, et al. Inexpensive anti-cysticercosis vaccine: S3Pvac expressed in heat inactivated M13 filamentous phage proves effective against naturally acquired Taenia solium porcine cysticercosis. Vaccine. 2008;26: 2899–2905. doi:10.1016/j.vaccine.2008.03.042

5. Ertel RL, Braae UC, Ngowi HA, Johansen MV. Assessment of a computer-based Taenia solium health education tool ‘The Vicious Worm’ on knowledge uptake among professionals and their attitudes towards the program. Acta Trop. 2017;165: 240–245. doi:10.1016/j.actatropica.2015.10.022

6. Huerta M, Aluja A De, Fragoso G, Vaccine AT-, 2001 U. Synthetic peptide vaccine against Taenia solium pig cysticercosis: successful vaccination in a controlled field trial in rural Mexico. Vaccines. 2001;20: 262–266. Available: https://www.sciencedirect.com/science/article/pii/S0264410X01002493

7. Jayashi CM, Kyngdon CT, Gauci CG, Gonzalez AE, Lightowlers MW. Successful immunization of naturally reared pigs against porcine cysticercosis with a recombinant oncosphere antigen vaccine. Vet Parasitol. 2012;188: 261–7. doi:10.1016/j.vetpar.2012.03.055

8. Sciutto E, Morales J, Martinez JJ, Toledo A, Villalobos MN, Cruz-Revilla C, et al. Further evaluation of the synthetic peptide vaccine S3Pvac against Taenia solium cysticercosis in pigs in an endemic town of Mexico. Parasitology. 2007;134: 129–133. doi:10.1017/S0031182006001132

9. Allan JC, Velasquez-Tohom M, Fletes C, Torres-Alvarez R, Lopez-Virula G, Yurrita P, et al. Mass chemotherapy for intestinal Taenia solium infection: Effect on prevalence in humans and pigs. Trans R Soc Trop Med Hyg. 1997;91: 595–598. doi:10.1016/S0035-9203(97)90042-0

10. Chung WC, Fan PC, Lin CY, Wu CC. Poor efficacy of albendazole for the treatment of human taeniasis. Int J Parasitol. 1991;21: 269–270.
