# Supplementary material for: A qualitative assessment of the context and enabling environment for the control of Taenia solium infections in endemic settings
Source: PLoS Negl Trop Dis. 2021 Jun 11;15(6):e0009470. doi: 10.1371/journal.pntd.0009470 (PMC8221787; doi:10.1371/journal.pntd.0009470)
Supplement: S2 Table — (DOCX) [file pntd.0009470.s003.docx]

**S2 Table: Studies focusing on testing effectiveness of control interventions**

| Study ID | **Target population and study site** | **Intervention** | **Features of study area at implementation** | **Conceptual framework/impact pathway** | **Methodology/ study design** | **Outcomes/findings** | **Challenges encountered and opportunities** | **Comments from KII** |
| --- | --- | --- | --- | --- | --- | --- | --- | --- |
| Garcia et al., 2016[1] | Tumbes, Peru; 10,753 humans and 17,102 pigs in phase 1 and 10,380 humans and 13,488 pigs and final scale up phase 3 107 villages, covering 81,170 humans and 55,638 pigs | Phase 1 screening of humans and pigs, antiparasitic treatment, prevention education,  and pig replacement in 42 villages for 1 yr. Phase 2 mass treatment with  mass screening and phase 3 mass treatment of humans along with the mass  treatment and vaccination of pigs in a region | multi-institutional effort; both ministries of agriculture and health were involved demonstrating one health approach; personnel from the ministries carried out the activities. Region was highly endemic | Determining feasibility of regional elimination; effect measured by detailed necropsy to detect pigs with live, non-degenerated  cysts capable of causing new infection after 1 year. | An entire region was covered in 3 phases with different interventions or modifications | incidence rate ratios, 0.78 [95% confidence interval  {CI}, 0.64 to 0.95] and 0.79 [95% CI, 0.65 to  0.97], respectively; 14 (36.8%; 95% CI, 21.8 to 54.0) of humans were still infected 2 weeks after mass treatment in phase 1, as assessed by ELISA for coproantigen detection plus stool microscopy. | it is feasible to interrupt *T. solium*  transmission on a regional scale, thereby preventing  human and porcine cysticercosis; The  reservoir of infection in the intermediate host  was eliminated in 105 of 107 villages through a  1-year attack phase; elimination not maintained. | Not interviewed |
| Hobbs et al., 2018[2] | Students from grades three to six at the Nyembe (Nyembe), Kondwelani (Chimvira) and Gunda (Herode) primary schools, Zambia. A total of 99 students participated in the three workshops: | Health education targeting school children | 90 % but open defection still practiced; 98% of the region’s estimated 66 000 pigs are reared under small-scale ‘backyard’ conditions; scavenging. No mention of stakeholders; no information on budget. delivery  of the educational component required only a laptop,  projector and small generator. | determine the impact education with the vicious worm program on knowledge uptake in primary school students, evaluate by change in knowledge from the baseline level ; no plans for scaling up | Pre and post intervention study | Post-questionnare. knowledge uptake of 11.5%. Increases of 10% or more were seen for 14 (82%) questions, and six (35%) increased by at least 25%. Increased knowledge was seen in seven of the eight QS1 categories (88%), with five (71%) increasing by 10% or more (P < 0.05). the key concepts for parasite control were better understood by the students after the educational workshops. | The differences in questionnaires used and the unavailability of individuals’ response data from the Nyembe workshop prevented a comprehensive comparison of knowledge and knowledge uptake across the three study groups on the individual level. | Important to understand sociocultural context; work with all stakeholders; build capacity local staff |
| O'neal et al., 2014[3] | Piura Province, Peru; 1058 residents in intervention village and 753 residents in control village= 1,811 people | Ring screening of people within 100 metres and treatment of positive ones with niclosamide | There was excellent participation of local community; Pigs are typically allowed to roam unrestrained in the village to forage as this reduces or eliminates owner investment in feed. pigs reared as a source of income and meat protein; there was high degree of community involvenment. | Treatment of people after ring screening and evaluation using incidence of exposure by sampling the pig population every 4  months for serum antibodies against cysticercosis using enzyme-linked immunoelectrotransfer blot. | a controlled prospective interventional cohort pilot study. We treated participants with suspected or confirmed taeniasis with a single oral dose of niclosamide according to their weight conduct mass treatment and screening for taeniasis | Over the entire study period the sero-incidence decreased 41% in the intervention community (incidence rate ratio [IRR] 0.59, 95% CI 0.41–0.87) and remained unchanged in the control village (IRR 1.01, 95% CI 0.70–1.47). There was 41% greater reduction in sero-incidence between baseline and study end in the intervention village compared to the control village (IRR 0.59, 95% CI 0.35–0.97). prevalence of taeniasis was nearly 4 times lower in the intervention village than in the control at study end (PR 0.28, 95% CI 0.08– 0.91). | ring-screening for taeniasis may reduce transmission of T. solium in a rural endemic area | Involve government and community. Hold open community meetings to sensitize community; work with other stakeholders including local NGOs. Collaboration was important |
| **Study ID** | **Target population and study site** | **Intervention** | **Features of study area at implementation** | **Conceptual framework/impact pathway** | **Methodology/ study design** | **Outcomes/findings** | **Challenges encountered and opportunities** | **Comments from KII** |
| Braae et al., 2016[4] | Mbozi and Mbeya district, Tanzania | School-based mass drug administration (MDA) of praziquantel was carried out based on schistosomiasis  Endemicity with a 36 months follow-up | village leaders, school headmasters, and head teachers involved in planning; there was high level of engagement between different stakeholders | Aim was to effects of the National Schistosomiasis Control Programme  on prevalence of taeniosis and porcine cysticercosis over a four-year period in Tanzania. Evaluation by prevalence of taeniosis and porcine cysticercosis by B158/B60 Ag-ELISA | multiple cross-sectional surveys carried out. MDA of praziquantel at 40 mg/kg to school-aged children 3 times in Mbozi district and 2 times in Mbeya district by the NSCP. Stool samples were collected in 14 villages from the human population | significant decrease in prevalence (13% to 8%) of porcine cysticercosis (p = 0.002, OR 0.49, CI: 0.32–0.76) was reported in Mbozi. 36% drop in prevalence of porcine cysticercosis Mbozi district and 23% drop in Mbeya. Prevalence of taeniosis had dropped from 4.1% to 1.8% | National Schistosomiasis Control Programme (NSCP) by the government of Tanzania provided support framework for the work; elimination would require a one health approach. Integration with other national control programs may be cost effective but other targeted approaches are needed | Important to work with different stakeholders. Maintaining visibility in the project area is important to ensure sustained interest by local communities |
| Alexander et al., 2011[5] | South India, Tamil Nadu, Kaniyambadi, a rural block | praziquantel, niclosamide and targeted therapy | The cost per person screened by stool testing for coproantigens was US $ 12, and the cost per case of taeniasis detected was US $ 4051, The cost per person screened was US $ 10.8; cost of hospitalization for the above step, which was US $ 30 per stool-positive subject in our study. | Aim was to evaluate the cost-effectiveness of three strategies for the control of taeniasis in a  community, in terms of cost per case treated. rate of taeniasis as detected by ELISA for coproantigen; evaluated by costs per case detected and treated for each and prevalence of taeniasis | screening of stool samples for coproantigens by ELISA and therapy for positive. 2^nd^ universal screening using only stool microscopy followed by targeted therapy of stool positive persons. The third option would be mass treatment with oral niclosamide | mass therapy without screening for taeniasis would be the most  economical strategy in terms of cost per case treated. For each strategy, costs per case treated are higher at low prevalence of  taeniasis, with a sharp rise below 15%. | Mass therapy without screening; This option may be more cost-effective per case of taeniasis treated than the others but the acceptability, feasibility and actual costs in India would have to be explored. Integration with other STH control programs would increase cost effectiveness. response rate for obtaining stool samples was low | Not interviewed |
| **Study ID** | **Target population and study site** | **Intervention** | **Features of study area at implementation** | **Conceptual framework/impact pathway** | **Methodology/ study design** | **Outcomes/findings** | **Challenges encountered and opportunities** | **Comments from KII** |
| Anna et al., 2016[6] | Phongsaly, in Mai District bordering Vietnam, Lao PDR. one village, 50/55 of permanent households (90.9%) households had at least one member eligible; over 75% of the eligible village pig population | Mass drug administration with a 3-day albendazole 400 mg to humans. At these times, and again in October 2014, eligible village pigs received TSOL18 vaccination and an oral dose of oxfendazole anthelmintic at 30 mg/kg, | local government medical personnel, project veterinary staff were involved in administering the MDA and monitoring. less than 20 percent have access to toilet, low-input production systems with many free ranging pigs. consumption of raw pork; village was not easily accessible during rainy season. | investigate T. solium control with treatment of both humans and pigs taeniasis prevalence were estimated via copro-antigen ELISA | A pilot study involving 2 rounds of community MDA with a 3- day albendazole 400 mg protocol, vaccination of pigs with the TSOL18 anti-cysticercosis vaccine and oral oxfendazole at 30 mg/kg | 9/138 cases of taeniasis. Taeniid eggs were detected in only one of the post-interventions copro-antigen positive. A 78.7% reduction to 6.52% (95% C.I.3.4–9.5%) accounting for clustering and a finite population; true village post intervention prevalence was returned as 0% (95% C.I. 0–5.1%), | A monitoring and evaluation of progress. lack of adequate facilities in northern Lao PDR for large-scale fine dissection of pigs, post-mortem analyses to identify cysts were not possible. Despite covering over 85% of the eligible human population at each MDA, this only related to approximately 60% of the total village population and compliance is a challenge | Important to consider context when designing and planning for interventions, involve local community especially for therapeutic interventions. Empower them to make decisions; work with both ministry of health and livestock; there was good government support |
| Ngowi et al., 2007[7] | People in 72 villages in Mbulu district Tanzania and evaluate in 42 villages | Health and pig management education intervention | Rural settings in Mbulu district, subsistence rearing of pigs as income source, no information on stakeholder involvement or policy environment | a financial benefit-cost analysis for the health education intervention, also effectiveness of the intervention reducing incidence rate of PCC; no plan for continuous reinforcement of the messages | Benefit-cost analysis of the intervention, effectiveness measured by reduced incidence of PCC by Ag-ELISA | Significant benefit of the intervention [NPV: US $3507 (95% CI: 3421 to 3591); IRR: 370%]. over 5 years, sensitivity analysis shows intervention will remain efficient over time, but follow-up was on for 4 months | Seasonality of feed availability led to drop out of 52% of baseline farmers, worry of outbreak of African Swine Fever in neighbouring regions; no mention of how they adjusted considering the challenges mentioned. | We have not been involving national stakeholders; no government funding; meat inspection guidelines exist but no meat inspectors; no policy on control of T. solium; superstitions in giving human samples |
| Ngowi et al., 2009[8] | People in 72 villages in Mbulu district Tanzania and 9 public health officials | health-promotion strategy-the PRECEDE-PROCEED model | Rural settings in Mbulu district, process evaluation applied, community assessment conducted to inform design, only extension and health officials mentioned | Health promotion model used to design and evaluate intervention, process evaluation applied; no plan for continuous reinforcement of the messages | Process evaluation applying the PRECEDE-PROCEED model, incidence of PCC by Ag-ELISA and KAPs evaluation | 20% reduction in consumption of cyst-infected pork, 43% reduction in incidence of PCC | Lack of holistic approach for example environmental health interventions, no public policy support; no attempt to involve other sectors in design and implementation. | Same as above |
| Ngowi et., 2011[9] | Farmers Iringa Rural and Chunya districts in Tanzania | Health education - training by a trained livestock extension officer, a video show, and distribution of a leaflet and a  booklet | Small scale pig rearing with sale to urban areas, extension worker and farmers mentioned as stakeholders, no mention of a baseline survey, only some terminology changed from the same messages used in another region | a quasi-experimental study design with pre- and post-intervention assessments of same  respondents to obtain paired data.  Change in KAPs taken to mean adoption; no plan for continuous reinforcement of the messages | Pre and post intervention of the same group | significant improvement in the knowledge and attitudes towards Taenia solium  control (P < 0.001), no change in consuming infected pork 28.4% of the respondents informed that they would still consume it | Few women attended the training raising questions of reach, authors acknowledge most pigs are reared by women; implementers did not adapt to overcome this challenge. Meat inspection laws should be improved; | Same as above |
| Ngowi et al., 2008[10] | Rural farmers in Mbulu district, Tanzania | Health education trial- video, leaflets, booklets, posters,  and a training manual | Baseline survey conducted; no mention of stakeholders involved included livestock/agricultural extension officers  (LFEO) and two health workers from psychiatry department and village leadership who helped identify target farmers | Measured effectiveness of health education by incidence of PCC and change in KAPs. Implementation process well explained; No plan for sustainability or scale-up of the intervention | a randomised community-controlled trial to measure effectiveness of health education using incidence of PCC by Ag-ELISA | incidence rate in the control group 10–12 months after the  intervention was 69 (95% CI: 65, 72) per 100 pig-years and 25 (95% CI: 23, 28) per 100 pig years, and intervention group was 44 (95% CI: 41, 47) per 100 pig-years and 12 (95% CI: 11, 14)  per 100 pig-years  using Ag-ELISA and lingual examination,  Knowledge on transmission improved >42%, consumption of infected pork reduced by 20% | livestock/agricultural extension officers and psychiatry department-local health workers not include in the farmers’ training (Phase 2) because they were scheduled for other official duties  at that time.  Small sample size at evaluation due to loss to follow up | Same as above |
| Pondja et al., 2012[11] | Ango´nia district, Mozambique | Oxfendazole (OXF) for PCC in pigs | Baseline survey on prevalence and KAPs; Free roaming pigs, Stakeholders involved;  private company -Bayer-South Africa provided the oxfendazole, community authorities and farmers, NGO: no involvement of local community in design and no policy environment info. | A randomized controlled field trial to evaluate effectiveness of OXF using prevalence, incidence using Ag-ELISA and viable cysts at necropsy; impact shown in the short run but no plans for scaling or sustainability in the study area | A randomized controlled field trial; evaluation by prevalence, incidence and viable cysts. | 66.7% of  controls positive, 21.4% of the T1 and 9.1% of the T2 pigs positive. Incidence  of PCC lower in treated pigs as compared to controls;  (OR = 0.14; 95% CI: 0.05–0.36) or at 9 months of age (OR = 0.05; 95% CI: 0.02–0.16) after OXF treatment. | Ag-ELISA not good for monitoring effectiveness because cysticercal antigen levels take long to disappear from circulation and may not detect brain cysts. | No interview |
| Sarti et al., 2000[12] | Atotonilco village in Morelos state in Mexico; one district, 87% of the 3007 population treated with praziquantel | mass treatment with praziquantel 5mg/kg as a single dose for Taeniasis | Field staff, laboratory staff, community members mentioned as stakeholders; 31% toilet coverage and improved to 64 after intervention, outdoor defecation reduced; baseline conducted; no mention of community involvement in design; no mention of working with MOH; improvement of sanitation unrelated to intervention. | Treatment of Taeniasis will ultimately reduce incidences of PCC and NCC; mass chemotherapy against taeniasis can have impact in the short and long term; Late-onset convulsive crisis and specific antibodies can indicate HCC and exposure; no plans for scaling | A population-based intervention study with baseline at T0 and evaluation by prevalence of taeniasis, NCC and PCC after 42 months | 11.3% p=0.85 change at T1 and 52.4 p=0.5 change by T2 by tongue palpation and 4=54.5% p= 0.05 change at T1 and 29.6% p=0.4 by ELISA of PCC; 56% by egg detection p=0.160 and 61.2% change by coproantigen p= 0.020 of Taeniasis; Late-onset general seizures decreased 70%. | Half dose 5mg/kg used to avoid exacerbation of neurocysticercosis but author notes this will increase direct costs; recommended is 10mg/kg; close supervision required in administration | No interview |
| Steinmann et al., 2011[13] | Menghai county/ Nongyang village in China; compliance  was estimated to be 80%. Recruitment was to be stopped once 400  individuals had been enrolled. 5 years and above | single-dose and triple-dose albendazole and mebendazole against soil-transmitted helminths and Taenia spp | Stakeholders; village head, village committee, and local health care officials, WHO regional office in Hanoi, Vietnam; no mention of involvement of target community in design; no mention of scaling | The intervention was not intended to evaluate effect on T. solium but STH as proxy; Kato-Katz thick smears- Eggs per gram of stool (EPG) and egg reduction rates (ERRs); An open-label trial design was adhered to due to the  complexities and high cost for implementing a double-blind trial  in a field setting; | a randomized controlled trial | 69% (95% CI: 55–81%) of hook worm infections cured with single dose of albendazole, while 31% (95% CI: 20–45%) was cured with triple dose. triple-dose albendazole cured significantly more hookworm infections (92%, 95% CI: 81–98%) than triple dose  mebendazole (58%, 95% CI: 46–71%). Single dose less  efficacious against *Tichuris trichiura* (mebendazole: 40%, albendazole: 34%., single dose of either drug cured half and triple dose cured all infection- triple dose albend. prevalece=o and 1.2 for triple dose mebend. | assessment of the locally endemic STHs, and the adaptation of the employed anthelminthic drug regimens to the prevailing situation emphasized; opportunity for integrating different interventions for several parasites demonstrated | Important to work with all actors – animal and public health; policy support important and align with programs working in the target area; integration is important and we demonstrated it can work; working with international organizations like WHO can help; meat inspection should be enforced; prepare practical messages for the health education intervention |
| Steinmann et al.,2015[14] | Nanwen upper,  Nanwen lower and Mangguo new villages in Menghai county in China; coverage rates around 80–90% of  the eligible population | health education and MDA with albendazole and latrine construction | Stakeholders; local village doctors and leaders, local construction company; no latrines but village had access to clean water; no mention of involvement of target community in design | Outcomes measures;  measure of prevalence of Taeniasis and EPG; target was also om other STHs; no mention of plans to maintain sustainability and scaling up. | a prospective community-based intervention study | per village for Taenia spp. Prevalence reduced by 54.7, 22.6 and 60.3% in Namwen upper, Namwen lower and Mangguo new village respectively; Only bi-annual treatment combined with latrine construction and health education significantly impacted on the prevalence of Taenia spp., | China government  subsidizes local infrastructure development,  an opportunity for similar projects; relaxation of intervention led to raise in prevalence of the STHs; MDA needed more frequently; different effects of the drug on different STHs; The rather high cost of about US$ 300 per latrine leading to sustainability issues | Same as above |
| Alexander et al., 2012[15] | Tamil Nadu State/Kaniyambadi, a rural development  Block in India; 120 households; 10 – 20 students from 3 high schools | Health education to school children and community | Pigs roamed free; One-third of the  houses in the area had toilets; drinking water was available; Implementation done by trained community volunteers and a qualified health educator; community not involved in design; senior social workers and doctors involved to evaluate the intervention; no mention of national stakeholders | Health education program to school children and community; evaluated through KAPs after 6 months; baseline survey was conducted; change in seroprevalence of PCC and taeniasis not evaluated; no mention of scale-up | A health education strategy designed based on baseline survey;messages were delivered through street plays,  songs, interactive discussions, posters, banners and handbills; study design not stated | a 46% increase in the overall score of knowledge and practices; washing hands with soap and water after using toilet improved by 3.6(23.7% to 86.4%), open defecation decreased by 23%; Awareness about the method of spread of taeniasis and cysticercosis improved by 3 times( from 10.5% to 28.6%) p<0.001 | Evaluation not done on same subjects; change in seroprevalence of PCC and Taeniasis not evaluated | Not interviewed |
| Ash et al., 2017[16] | Phongsaly village 700km north of Vientiane LAO PDR; 300 people in 60 households; 64% coverage; n=146/298 received 3 doses in MDA1 and n=173/293 received 3 doses in MDA2; 108 loss to follow-up | MDA with albendazole 400mg/kg over 3 consecutive days | Low sanitation, open defecation, subsistence pig rearing; consumption of raw and/or undercooked pork, practiced; Some stakeholders; Lao PDR Ministry of Health Department for Communicable Disease Control, Department of Hygiene and Prevention, and the Neglected Tropical Diseases administered drug and monitored; baseline survey conducted. | Reduce prevalence of taeniasis and other helminths using one health approach; 2 rounds of MDA with 400mg/kg albendazole – has broad spectrum; after 5 months | A before and after study; McMaster technique used for STH and molecular techniques for T. solium and hookworms | after MDA1 overall 66% P < 0.0001). prevalence reduction for individual parasite species for A. lumbricoides (95.6%; P < 0.0001), hookworm (83.4%; P < 0.0001), Taenia spp. (79.4%; P = 0.012), T. trichiura (69.2%; P < 0.0001). After MDA2 Overall parasite prevalence 20.8%, greatest reduction in individual parasite species was detected for Taenia spp. (100%) | Existence of school deworming programme may have compromised compliance for children; varying proportion of unidentified faecal samples were obtained in the study; eligibility and compliance of community members, and the level of re-infection which is attained post treatment were challenges; need for integration | We worked with ministry of health and ministry of agriculture but depends on the intervention whether pigs or humans; STH and T. solium have high priority in LAO; involving local community will ensure sustainability. |
| Assana et al., 2010[17] | Mayo-Danay administrative department in Cameroon; 120 piglets pairs-vaccinated and not vaccinated | Vaccination of pigs with TSOL18 and administration of oxfendazole | Pigs roam free during dry season; more than 40% of houses that keep pigs do not have  latrines; baseline conducted through tongue palpation; no mention of stakeholders; no involvement of local community in design | Vaccination to protect uninfected pigs and oxfendazole to treat infected pigs; Assessment by prevalence and number of viable cysts and ELISA for antibody titres; provide proof-of-principle; no mention of scaling | A field trail for TSOL18 vaccine; vaccinate piglets at 2–3 months of age and give a booster immunization 4 weeks later. At the time of the second immunization, the pigs were given oxfendazole | Reduction in the prevalence of infection from 19.6% (19/97) in paired control pigs to 0% (0/97) in paired vaccinated pigs (P < 0.0001). Control group had 20% prevalence | Combined application of TSOL18 vaccination and a single  oxfendazole treatment has potential to eliminate T. solium | Not interviewed |
| Beam et al., 2018[18] | Piura province, Peru; 1,250 pigs in the cohort including 615 (49.2%) in the intervention  villages and 635 (50.8%) in the control | Household- and school-based  education about the parasite life cycle and methods to  prevent infection were offered in all villages | 67.5% latrine coverage; free roaming pigs Stakeholders: community health workers, health post, residents, school children, key actors (political and religious  authorities, pig farmers and vendors, ministry of health office of epidemiology; | study outcome was porcine sero-incidence  measured every 4 months; secondary outcomes included the  prevalence of porcine cysticercosis and human taeniasis at study  end; surveillance and reporting system was also tested | prospective trial; A local surveillance  and response system was established in intervention villages  along with a campaign to promote reporting of infected pigs | no difference in the sero-incidence after 12 months in the  intervention versus control villages | There was some level of community involvement | Not interviewed |
| Beam et al., 2019[19] | Peru; Workshop participation of at least 1 adult per household was 41/84  (49%), 25/34 (74%), 29/68 (43%), and 15/49 (31%) per village in the 4 villages | Health education | Context not reported; no mention of stakeholders; volunteers involved; | workshop included presentation of local economic  and epidemiologic data, followed by hands-on participation in pig dissection, group discussion of the T. solium life  cycle, and viewing of eggs and nascent tapeworms with light microscopes; use of local evidence and experiential learning positively affected knowledge | Before and after study | Knowledge of human-to-pig transmission increased by 38%(P< 0.01) for attendees and 23%(P< 0.01) for non-attendees, knowledge of pig-to-human transmission increased by 42% (P < 0.01) for workshop attendees and 9% (P < 0.05) for non-attendees, Only workshop attendees had gains in knowledge of human-to-human transmission (12%, P < 0.05 versus 5%, P = 0.13) | Discussing control interventions with local community can help in design | Not interviewed |
| Camacho et al.,1991[20] | La Curva, Navolato in the state of Sinaloa in Mexico; Over 70% of the population over five years of age | large scale treatment of the population with praziquantel at 10mg/kg | 25% sanitation; free roaming and tethering of pigs; no mention of scalability but praziquantel recommended | Evaluation of the intervention in people was by measuring prevalence of PCC in pigs and Taeniasis by microscopy and ELISA | census, stool sampling and blood from humans and inspection of pig by tongue palpation, treatment with praziquantel and then evaluation after 1 year | from 1.32% of taeniasis at baseline and no case at end line (0%); Relative risk decreased from 2.95 (95% CI = 1.56-5.56) before treatment to 0.85 (95% CI = 0.2- 3.4) after treatment. | There was administration of other antiparasitic drugs in the same area for other STHs; there could have been complication in people with NCC because of the dose of praziquantel used. | Not interviewed |
| Kabululu et al., 2018[21] | Mbozi and Mbeya Rural districts in Tanzania; 92, 51 and 78 pigs during baseline, first follow-up and second follow-up rounds respectively, which is 16.4%  of all eligible pigs in the selected households | Oxfendazole for T. solium and ivermectin for other nematodes and animal husbandry education; | Majority semi-confined pigs; District Livestock Office, local administrative leaders, Farmers, local mason/  carpenter, extension officers Oxfendazole donated by private company; a baseline was conducted | integrated intervention in the control of endo- and  ectoparasites with oxfendazole for specifically T. solium; evaluation by Ag-ELISA,  McMaster faecal egg counting technique and body searches/skin scrapings; no mention of plans for scaling up; sustainability was a problem – reduced compliance | A repeated cross-sectional group randomization design was used; specific training and technology transfer of improved pig pens, improved pig feeds and feeding practices, and treatment with OFZ and IVM; control group received ivermectin treatment only; 3 model pens constructed; 2 follow-ups 7 months apart each. | 12.6% in intervention and 9.8% in control;59.3% for intervention and 70.4 in control for GI helminths; no significant change p= 0.429. Fluctuations in sero-prevalence of PC were observed in both groups where prevalence increased from baseline to first follow-up and declined from first to second follow-up; significant difference between the two groups in change of prevalence of T. suis | underestimation because serology is not able to provide quantitative data; reduced compliance as this was a field study | Not interviewed |
| Mwidunda et al., 2015[22] | Mbulu district, Tanzania; 60  schools (30 primary, 30 secondary) in Mbulu district. | Health education | School children and teachers were involved. | Health education to school children evaluated 12 months with assessments by KAPs immediately, at 6 months and at 12 months; training school children in the hope that they will pass the knowledge to the community. | A cluster randomised controlled health education intervention trial | The intervention improved knowledge about human cysticercosis the most, followed by that about human tapeworm, epilepsy and porcine cysticercosis. The intervention  improved the total score by about 9–10%. decreased to 3.2% (95% BCI: 2.2%-4.3%) after 6 months. For secondary school student’s knowledge was sustained over 12 months | large variations in the baseline knowledge and attitudes regarding T.  solium cysticercosis and taeniasis in several variables tested among schools | Not interviewed |
| Garcia et al., 2006[23] | Huancayo, Quilcas district in Peru; 5,658 resident individuals and 716 pigs; coverage was 75%, ranging from 69% to 80%. | MDA with praziquantel in humans and oxfendazole in pigs | key village leaders, village leaders consulted the community on willingness to participate, sensitization to groups of villagers conducted | Outcomes were evaluated every 4 months after the  intervention for a total of 20 months; Only through active  participation of peasants it can be expected that an intervention program be sustained, and educational programs be accepted. Evaluation by comparing incidence rates (seroconversion in pigs who were seronegative 4 months before) in treatment versus control villages | The control intervention consisted of one round of mass chemotherapy for intestinal tapeworm infection in humans with a single dose of 5 mg/kg of praziquantel and two rounds of mass chemotherapy for porcine cysticercosis with a single dose of 30 mg/kg of oxfendazole | clear effect in decreasing prevalence (odds ratio, 0.51; P < 0.001) and incidence (odds ratio, 0.39; P0.013)  in the treatment area after the intervention | very high proportion of pigs were sampled, principally because hog cholera vaccines and veterinary  attention gained the support of the population and sustained it throughout the study. There were only a few refusals  from cases where an animal coincidentally died soon after a sampling campaign and the family attributed the death of the animal to the blood sampling |  |
| Cruz et al., 1990[23] | Gonzanama and Catamayo in Loja and Balsas and Marcabelli in El Oro provinces, Ecuador; total of 10173 people treated;12.3% of total population treated | Population based treatment of taeniasis with praziquantel | provincial authorities  and local community councils or leaders consulted in organization of the study; National University in Loja- provided 64 auxiliary staff, as follows: seven physicians, two veterinarians, one  public health specialist, 26 students, three social workers, seven sanitary inspectors, six teachers, 9 volunteers, and three drivers;  Praziquantel donated by private company; local committee; a lot of sensitization was done through local radio and posters | Aim was on how to operationalize T. solium control by using praziquantel evaluated by Kato-Katz technique and pigs by necropsy; community showed interest for sustainability | 3 phases; weighed and given a single dose of praziquantel 5 mg/kg. (90%) houses revisited during the 48-72 hours after treatment and questioned about any side-effects and whether they had expelled tapeworms. second treatment of 739 people (539 of whom had previously been treated and 200 of whom were treated for the first time) | 1.6% meaning 0% prevalence at end line; a single dose of praziquantel  (about 5mg/kg body weight) is effective | Long term evaluation needed; 90% of people wanted project to continue | Not interviewed |
| Gonzales et al., 2001[24] | Casacancha, Peru | single dose oxfendazole treatment for pigs | cysticercosis endemicity and long-term  successful collaboration with villagers during previous surveys. | Evaluated eight weeks after treatment by checking acquisition of cysts (viable or degenerated) or seroconversion  by EITB. Aim was to see if pigs with cysticercosis  can acquire new infections after being treated with OFZ. | an experimental study pigs were treated with a single oral dose of 30 mg/kg of OFZ given as a veterinary aqueous suspension. Treated pigs were kept here for eight weeks after treatment | New infections were detected by serology in 15 (47%) of 32 control pigs, and by the presence of cysts in 12 (38%) of 32. Among these 12 pigs, viable cysts were found in seven carcasses; efficacy of OFX shown | One treated and nine control pigs were not recovered because of various reasons, mostly because villagers sold or slaughtered them for consumption. A minor drawback in the use of OFZ is that some cysts may survive in the pig’s brain after treatment. | not interviewed |
| Keilbach et al., 1989[25] | Los sauces village, Guerrero, Mexico; 900 individuals received praziquantel, 60 % coverage; follow up after 4 months for pigs and 1 year for change in KAPs after education | health education and praziquantel and niclosamide for those with NCC; education delivered through meetings, lectures and demonstrations; | Worked with local research institute and university who provided technicians, nurses and minimum laboratory facilities; pigs are slaughtered at home and sold to village butcheries. Sometimes loaded into trucks and transported to other villages. No meat inspection | Evaluation of the intervention by prevalence by tongue palpation and ELISA and KAPS; Behaviour and attitude did not change after 2 years; knowledge improved slightly; | sensitization, coprological examination and ELISA, treatment with 5mg praziquantel and niclosamide for people with NCC, tongue examination of 200 pigs and ELISA, examination of soil samples | 11% of pigs were positive at the final end of the evaluation by tongue palpation. Prevalence increased; in humans prevalence reduced from 3 to 0 %; after 2 years 2% of adults and 76% of children understood life cycle of taenia solium and cause of HCC in humans and PCC in pigs | illiteracy levels were high, and people did not want give up their traditional ways; taeniasis and HCC not appreciated as a major health problem,low percentage of treated people, open defaction and continued consumption of infected meat explain the increase prevalence of PCC. | Not interviewed |

**References**

1. Garcia HH, Gonzalez AE, Tsang VCW, O’Neal SE, Llanos-Zavalaga F, Gonzalvez G, et al. Elimination of *Taenia solium* Transmission in Northern Peru. N Engl J Med. 2016;374: 2335–2344. doi:10.1056/NEJMoa1515520

2. Hobbs EC, Mwape KE, Van Damme I, Berkvens D, Zulu G, Mambwe M, et al. Preliminary assessment of the computer-based *Taenia solium* educational program ‘The Vicious Worm’ on knowledge uptake in primary school students in rural areas in eastern Zambia. Trop Med Int Heal. 2018;23: 306–314. doi:10.1111/tmi.13029

3. O’Neal SE, Moyano LM, Ayvar V, Rodriguez S, Gavidia C, Wilkins PP, et al. Ring-Screening to Control Endemic Transmission of Taenia solium. PLoS Negl Trop Dis. 2014;8. doi:10.1371/journal.pntd.0003125

4. Braae UC, Magnussen P, Harrison W, Ndawi B, Lekule F, Johansen MV. Effect of National Schistosomiasis Control Programme on Taenia solium taeniosis and porcine cysticercosis in rural communities of Tanzania. Parasite Epidemiol Control. 2016;1: 245–251. doi:10.1016/j.parepi.2016.08.004

5. Alexander A, John KR, Jayaraman T, Oommen A, Venkata Raghava M, Dorny P, et al. Economic implications of three strategies for the control of taeniasis. Trop Med Int Health. 2011;16: 1410–1416. doi:10.1111/j.1365-3156.2011.02850.x

6. Okello AL, Thomas L, Inthavong P, Ash A, Khamlome B, Keokamphet C, et al. Assessing the impact of a joint human-porcine intervention package for Taenia solium control: Results of a pilot study from northern Lao PDR. Acta Trop. 2016;159: 185–191. doi:10.1016/j.actatropica.2016.03.012

7. Ngowi HA, Carabin H, Kassuku AA, Mlozi MRS, Mlangwa JED, Willingham AL. A health-education intervention trial to reduce porcine cysticercosis in Mbulu District, Tanzania. Prev Vet Med. 2008;85: 52–67. doi:10.1016/j.prevetmed.2007.12.014

8. Ngowi HA, Mlangwa JE, Mlozi MR, Tolma EL, Kassuku AA, Carabin H, et al. Implementation and evaluation of a health-promotion strategy for control of Taenia solium infections in northern Tanzania. Int J Heal Promot Educ. 2009;47: 24–34. doi:10.1080/14635240.2009.10708154

9. Ngowi HA, Mkupasi EM, Lekule FP, Willingham IL, Thamsborg SM. Impact of farmer education on their knowledge, attitudes, and practices in southern Tanzania: A case for Taenia solium control. Livest Res Rural Dev. 2011;23: 1–7.

10. Ngowi HA, Mlangwa JED, Carabin H, Mlozi MRS, Kassuku AA, Kimera SI, et al. Financial efficiency of health and pig management education intervention in controlling porcine cysticercosis in Mbulu District, northern Tanzania. Livest Res Rural Dev. 2007;19: 19062.

11. Pondja A, Neves L, Mlangwa J, Afonso S, Fafetine J, Willingham AL 3rd, et al. Use of oxfendazole to control porcine cysticercosis in a high-endemic area of Mozambique. PLoS Negl Trop Dis. 2012;6: e1651. doi:10.1371/journal.pntd.0001651

12. Sarti E, Schantz PM, Avila G, Ambrosio J, Medina-Santillán R, Flisser A. Mass treatment against human taeniasis for the control of cysticercosis: A population-based intervention study. Trans R Soc Trop Med Hyg. 2000;94: 85–89. doi:10.1016/S0035-9203(00)90451-6

13. Steinmann P, Utzinger J, Du Z-W, Jiang J-Y, Chen J-X, Hattendorf J, et al. Efficacy of single-dose and triple-dose albendazole and mebendazole against soil-transmitted helminths and Taenia spp.: a randomized controlled trial. PLoS One. 2011;6: e25003. doi:10.1371/journal.pone.0025003

14. Steinmann P, Yap P, Utzinger J, Du Z-W, Jiang J-Y, Chen R, et al. Control of soil-transmitted helminthiasis in Yunnan province, People’s Republic of China: experiences and lessons from a 5-year multi-intervention trial. Acta Trop. 2015;141: 271–280. doi:10.1016/j.actatropica.2014.10.001

15. Alexander AM, Mohan VR, Muliyil J, Dorny P, Rajshekhar V. Changes in knowledge and practices related to taeniasis/cysticercosis after health education in a south Indian community. Int Health. 2012;4: 164–169. doi:10.1016/j.inhe.2012.04.003

16. Ash A, Okello A, Khamlome B, Inthavong P, Allen J, Thompson RCA. Controlling Taenia solium and soil transmitted helminths in a northern Lao PDR village: Impact of a triple dose albendazole regime. Acta Trop. 2017;174: 171–178. doi:10.1016/j.actatropica.2015.05.018

17. Assana E, Kyngdon CT, Gauci CG, Geerts S, Dorny P, De Deken R, et al. Elimination of Taenia solium transmission to pigs in a field trial of the TSOL18 vaccine in Cameroon. Int J Parasitol. 2010;40: 515–519. doi:10.1016/j.ijpara.2010.01.006

18. Beam M, Spencer A, Fernandez L, Atto R, Muro C, Vilchez P, et al. Barriers to participation in a community-Based program to control transmission of Taenia solium in Peru. Am J Trop Med Hyg. 2018;98: 1748–1754. doi:10.4269/ajtmh.17-0929

19. Beam M, Spencer AG, Atto R, Camizan R, Vilchez P, Muro C, et al. To really know the disease: Creating a participatory community education workshop about taenia solium focused on physical, economic, and epidemiologic evidence. Am J Trop Med Hyg. 2019;100: 1490–1493. doi:10.4269/ajtmh.18-0939

20. Camacho SPD, Ruiz AC, Peraza VS, Ramos MLZ, Medina MF, Lozano R, et al. Epidemiologic study and control of Taenia solium infections with praziquantel in a rural village of Mexico. Am J Trop Med Hyg. 1991;45: 522–531. doi:10.4269/ajtmh.1991.45.522

21. Kabululu ML, Ngowi HA, Kimera SI, Lekule FP, Kimbi EC, Johansen MV. Effectiveness of an integrated intervention in the control of endo- and ectoparasites of pigs kept by smallholder farmers in Mbeya rural and Mbozi districts, Tanzania. Vet Parasitol Reg Stud Reports. 2018;13: 64–73. doi:10.1016/j.vprsr.2018.03.009

22. Mwidunda SA, Carabin H, Matuja WBM, Winkler AS, Ngowi HA. A school based cluster randomised health education intervention trial for improving knowledge and attitudes related to Taenia solium cysticercosis and taeniasis in Mbulu district, northern Tanzania. PLoS One. 2015;10: e0118541. doi:10.1371/journal.pone.0118541

23. Garcia HH, Gonzalez AE, Gilman RH, Moulton LH, Verastegui M, Rodriguez S, et al. Combined human and porcine mass chemotherapy for the control of T. solium. Am J Trop Med Hyg. 2006;74: 850–855. doi:10.4269/ajtmh.2006.74.850

24. Gonzalez AE, Gavidia C, Falcon N, Bernal T, Verastegui M, Garcia HH, et al. Protection of pigs with cysticercosis from further infections after treatment with oxfendazole. Am J Trop Med Hyg. 2001;65: 15–18. doi:10.4269/ajtmh.2001.65.15

25. Keilbach NM, de Aluja AS, Sarti-Gutierrez E. A programme to control taeniasis-cysticercosis (T. solium): experiences in a Mexican village. Acta Leiden. 1989;57: 181–189.
