# Supplementary material for: A qualitative assessment of the context and enabling environment for the control of Taenia solium infections in endemic settings
Source: PLoS Negl Trop Dis. 2021 Jun 11;15(6):e0009470. doi: 10.1371/journal.pntd.0009470 (PMC8221787; doi:10.1371/journal.pntd.0009470)
Supplement: S3 Table — (DOCX) [file pntd.0009470.s004.docx]

**S3 Table: Studies focusing on implementation research for control interventions or scale-up of interventions**

| **Study ID** | **Target population and study site** | **Intervention** | **Features of study area at implementation** | **Conceptual framework/impact pathway** | **Methodology/ study design** | **Outcomes/findings** | **Challenges encountered and opportunities** | **Comments from KII** |
| --- | --- | --- | --- | --- | --- | --- | --- | --- |
| Braae et al., 2017[1] | Mbeya and Mbozi district, Tanzania; 34% School age children in 14 villages | MDA with praziquantel | 54, 46, and 38% of the sampled people had no access to toilet Stakeholders; village leaders, school headmasters and head teachers. National Schistosomiasis Control Programme (NSCP), district health officer and the medical doctor; school-based MDA with praziquantel is carried out as part of the National Schistosomiasis Control Programme (NSCP) in schistosomiasis endemic districts part of support programs | MDA to school-aged children (SAC) combined with ‘track and treat’ of taeniosis cases in the general population; being in the community for extended time helped maintain support. | Three cross-sectional population-based surveys were performed in 2012 (R0), 2013 (R1), and 2014 (R2). ‘Track and treat’ for positive individuals | a drop in infection at R1 (P < 0.001, OR 0.49, CI: 0.32–0.74)twelve months after the MDA in both districts and at R2 ten months after the second round of MDA in Mbozi and 22 months after the first MDA (P < 0.001, OR 0.38, CI: 0.22–0.62); in some cases prevalence did not change | The persistence of infections means one health approach is needed; engaging community over long time helped support activities during implementation | Guidelines on meat inspection exist but not enforced; during implementation maintain visibility in the community; engage community and explain project |
| Bulaya et al., 2015[2] | Katete District, Zambia; 9 villages in Katete district | Community led total sanitation (CLTS) to control *Taenia solium* cysticercosis | open defecation practiced; free roaming pigs; Stakeholders; Ministry of Local Government and Housing, trained CLTS champions, local district veterinary authorities and local leaders; CLTS implemented by national government and Unicef | Improvement of sanitation will lead to reduced taeniasis and PCC prevalence; A trainer of trainers (ToT) workshop was conducted with District Council employees on how to train CLTS champions and trigger. PCC examined by B158/B60 Ag-ELISA after 8 mon. | A comparative research was conducted with pre and post-intervention assessments | Prevalence of PCC 13.5% and 16.4% after the intervention; At baseline, 29.1% of the respondents were unaware of pig cysticercosis com-pared to 56.8% at post-intervention. latrine use was at 41 (93.2%) at baseline and 62(84.9%) post-intervention; 32 new latrines constructed | The validity due to possibility of cross reactions with *T. hydatigena* metacestodes; an anthropological study provided insights; availability of women for the sessions due to seasonality; longer monitoring period needed | Many stakeholders were involved because it had backing of national govt.;local communities and leadership need to be involved more; no national policy on control of *T. solium* |
| Carabin et al., 2018[3] | Boulkiemdé, Sanguié, and Nayala provinces, Burkina Faso; 60 villages- 80 households, 60 participants in each village; 522 participants in intervention; 513 in control | community-based educational programme; intervention developed using PRECEDE-PROCEED; | 13.5% use toilet; pigs confined but fed feaces in one province village chiefs and household chiefs in planning; Water and Sanitation for Africa (WSA); development of the intervention  cost US$31 538; intervention was low-cost, culturally appropriate intervention; intervention developed in a participatory way; | Evaluation of the intervention by incidence and prevalence of  human *Taenia solium* cysticercosis by B158/B60 Ag-ELISA; 39/705 (5·5%) new toilets; increase community self-efficacy through a Self-esteem, Associative strengths, Resourcefulness, Action planning,  Responsibility (SARAR) approach via the Participatory Hygiene and Sanitation Transformation (PHAST) model | a cluster-randomised controlled trial | decrease (adjusted prevalence proportion  ratio 0·84, 95% CrI 0·59–1·18) to post-randomization cumulative incidence (adjusted  cumulative incidence ratio 0·65, 95% CrI 0·39–1·05) | weaker to no effects were observed when the smaller database of analytical  sample 1 was used. Implementation research required going forward | Not interviewed |

**References**

1. Braae UC, Magnussen P, Ndawi B, Harrison W, Lekule F, Johansen MV. Effect of repeated mass drug administration with praziquantel and track and treat of taeniosis cases on the prevalence of taeniosis in Taenia solium endemic rural communities of Tanzania. Acta Trop. 2017;165: 246–251. doi:10.1016/j.actatropica.2015.10.012

2. Bulaya C, Mwape KE, Michelo C, Sikasunge CS, Makungu C, Gabriel S, et al. Preliminary evaluation of Community-Led Total Sanitation for the control of Taenia solium cysticercosis in Katete District of Zambia. Vet Parasitol. 2015;207: 241–8. doi:10.1016/j.vetpar.2014.12.030

3. Carabin H, Millogo A, Ngowi HA, Bauer C, Dermauw V, Koné AC, et al. Effectiveness of a community-based educational programme in reducing the cumulative incidence and prevalence of human Taenia solium cysticercosis in Burkina Faso in 2011–14 (EFECAB): a cluster-randomised controlled trial. Lancet Glob Heal. 2018;6: e411–e425. doi:10.1016/S2214-109X(18)30027-5
